# The HCoV-HKU1 N-Terminal Domain Binds a Wide Range of 9-*O*-Acetylated Sialic Acids Presented on Different Glycan Cores

**DOI:** 10.1021/acsinfecdis.4c00488

**Published:** 2024-10-12

**Authors:** Ilhan Tomris, Anne L. M. Kimpel, Ruonan Liang, Roosmarijn van der Woude, Geert-Jan P. H. Boons, Zeshi Li, Robert P. de Vries

**Affiliations:** † Department of Chemical Biology & Drug Discovery, Utrecht Institute for Pharmaceutical Sciences, Utrecht University, Utrecht 3584 CG, The Netherlands; ‡ Complex Carbohydrate Research Center, University of Georgia, 315 Riverbend Road, Athens, Georgia 30602, United States

**Keywords:** HCoV-HKU1, coronavirus, glycans, sialic acid, multivalency, ligand, receptor binding

## Abstract

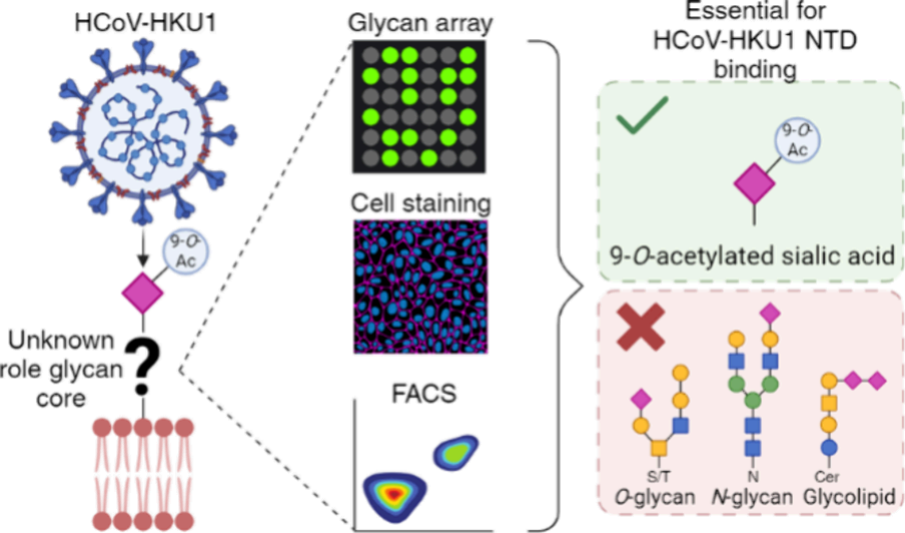

Coronaviruses (CoVs) recognize a wide array of protein and glycan receptors by using the S1 subunit of the spike (S) glycoprotein. The S1 subunit contains two functional domains: the N-terminal domain (S1-NTD) and the C-terminal domain (S1-CTD). The S1-NTD of SARS-CoV-2, MERS-CoV, and HCoV-HKU1 possesses an evolutionarily conserved glycan binding cleft that facilitates weak interactions with sialic acids on cell surfaces. HCoV-HKU1 employs 9-*O*-acetylated α2-8-linked disialylated structures for initial binding, followed by TMPRSS2 receptor binding and virus–cell fusion. Here, we demonstrate that the HCoV-HKU1 NTD has a broader receptor binding repertoire than previously recognized. We presented HCoV-HKU1 NTD Fc chimeras on a nanoparticle system to mimic the densely decorated surface of HCoV-HKU1. These proteins were expressed by HEK293S GnTI^–^ cells, generating species carrying Man-5 structures, often observed near the receptor binding site of CoVs. This multivalent presentation of high mannose-containing NTD proteins revealed a much broader receptor binding profile compared to that of its fully glycosylated counterpart. Using glycan microarrays, we observed that 9-*O*-acetylated α2-3-linked sialylated LacNAc structures are also bound, comparable to OC43 NTD, suggesting an evolutionarily conserved glycan-binding modality. Further characterization of receptor specificity indicated promiscuous binding toward 9-*O*-acetylated sialoglycans, independent of the glycan core (glycolipids, *N-* or *O*-glycans). We demonstrate that HCoV-HKU1 may employ additional sialoglycan receptors to trigger conformational changes in the spike glycoprotein to expose the S1-CTD for proteinaceous receptor binding.

Coronaviruses (CoVs) cause respiratory and digestive disorders, manifesting in various species, including dogs, cats, cattle, and other animals.^1^ These CoVs are separated into four genera based on their genomic structure and phylogenetic branching: Alpha (α), Beta (β), Gamma (γ), and Delta (δ), with many unidentified CoVs circulating in the natural reservoir (bats and rodents).^2^ Several α- and β-genus CoVs (Table S1) have crossed the human barrier and may cause mild to severe respiratory tract infections. These human coronaviruses (HCoVs) differ in receptor recognition, virus internalization pathways, and accessory genes. Receptor binding is driven by the spike (S) glycoprotein, which is displayed on the viral membrane and consists of two functional subunits: S1 and S2. The S1 facilitates cellular receptor interaction and is divided into the N-terminal domain (S1-NTD) and the C-terminal domain (S1-CTD). The binding of CoVs to specific receptors defines tropism, and both the S1-NTD and S1-CTD may function as the receptor binding domain (RBD).^1,3^ The S1-NTDs of CoVs contain an evolutionarily conserved galectin fold that functions as a (sialo)glycan-binding viral lectin,^4^ while the S1-CTD interacts with proteinaceous receptors. Following conformational rearrangement of S2, initiated by S1-receptor binding, membrane fusion is triggered, and viral entry occurs through the plasma membrane or endosomal pathway.^5^

The sugar layer covering eukaryotic cells, the glycocalyx, serves as a barrier against pathogens while simultaneously serving as an attachment site for these pathogens. Viral attachment is a crucial and dynamic process in host–cell infections, and several pathogens use sialoglycans as an initial attachment point,^6^ an important determinant for tropism and defining the host range.^7,8^ Sialylated glycans are employed by several (human) CoVs, albeit further modifications of the sialic acid (Sia) influence receptor attachment.^9,10^ Glycan structures with terminal Sia (α2-3-, α2-6-, and α2-8-linked) exist in several modified forms.^11^ These commonly occur on carbon 4, 5, 7, 8, and 9 positions, with regulation being dependent on physiological conditions and animal species.^7,9^ The *O*-acetyl modification of Sia is introduced in the Golgi apparatus by Sia-specific *O*-acetyltransferases (SOATs), with CAS1 domain containing 1 (CASD1) being the only SOAT identified so far.^12^ The exact role of *O*-acetylation is not well defined, and the abundance of this modification appears to differ per tissue.^7,8^ SARS-CoV-2, Bovine CoV (BCoV), HCoV-OC43, and HCoV-HKU1 engage 9-*O*-acetylated Sias using their S1-NTD.^13−16^ Interestingly, although these CoVs belong to the β-CoV genus, they do not cluster in the corresponding subgenera. SARS-CoV-2 belongs to the Sarbecovirus subgenus, while BCoV, HCoV-OC43, and HCoV-HKU1 are Embecoviruses,^17^ suggesting that 9-*O*-acetylated Sia recognition is an evolutionarily conserved property between CoVs.^13,16,18^ HCoV-OC43 and HCoV-HKU1 show high sequence similarity and recognize the same receptor; however, these viruses were introduced and adapted to the human population independently.^19,20^ HCoV-OC43 and HCoV-HKU1 S1-NTD both recognize 9-*O*-acetylated α2-8-linked disialic acid, while HCoV-OC43 displays promiscuity toward 9-*O*-acetylated α2-3- and α2-6-linked Sia.^10,16^ Recently, binding of the HCoV-HKU1 S1-NTD to a 9-*O*-acetylated sialic acid was shown to switch the HCoV-HKU1 RBD to an open state upon which binding with the proteinaceous receptor transmembrane serine protease 2 (TMPRSS2) can take place.^21−24^

Virus–glycan interactions are inherently of low binding affinity, requiring a multivalent display for receptor characterization. Additionally, multivalent ligands can mediate receptor clustering,^25^ which promotes virus–cell binding.^26^ Multivalent effects can be attained with precomplexation using antibodies.^27,28^ Furthermore, virus-like particles (VLPs) and nanoparticles (NPs) highly resemble pathogens by presenting antigenic epitopes in a highly dense and ordered array, enabling multiple binding events.^29,30^ In immunization and vaccine development, these multivalent molecules are favored compared to monovalent equivalents.^31^ Similarly, to increase the sensitivity through an increase in avidity, HCoV-HKU1 S1-NTD was presented on a self-assembling 60-mer to probe for sialoglycan binding and receptor specificity. In addition, expressing sialic acid-binding proteins with complete glycosylation may result in aggregation and dim binding signals, as previously observed for the hemagglutinin protein of the influenza A virus.^32,33^ To overcome the negative effect of complex glycosylation in receptor binding, we employed *N*-acetylglucosaminyltransferase I (GnTI)-deficient cells^34^ to assess the possible broader receptor specificities of HCoV-HKU1 S1-NTD. Here, we elaborate that multivalent presentation enabled HCoV-HKU1 S1-NTD to bind to the more abundant 9-*O*-acetylated α2-3-linked Sia-containing epitopes. Although HCoV-HKU1 and HCoV-OC43 are separated by considerable phylogenetic distance, glycan binding of HCoV-HKU1 is thus nearly identical to that of HCoV-OC43,^10,15^ indicating that the mode of receptor binding is conserved between embecoviruses.^15^

## Results

### Glycosylation and Multivalency in Virus–Receptor Interaction

**Figure 1:**
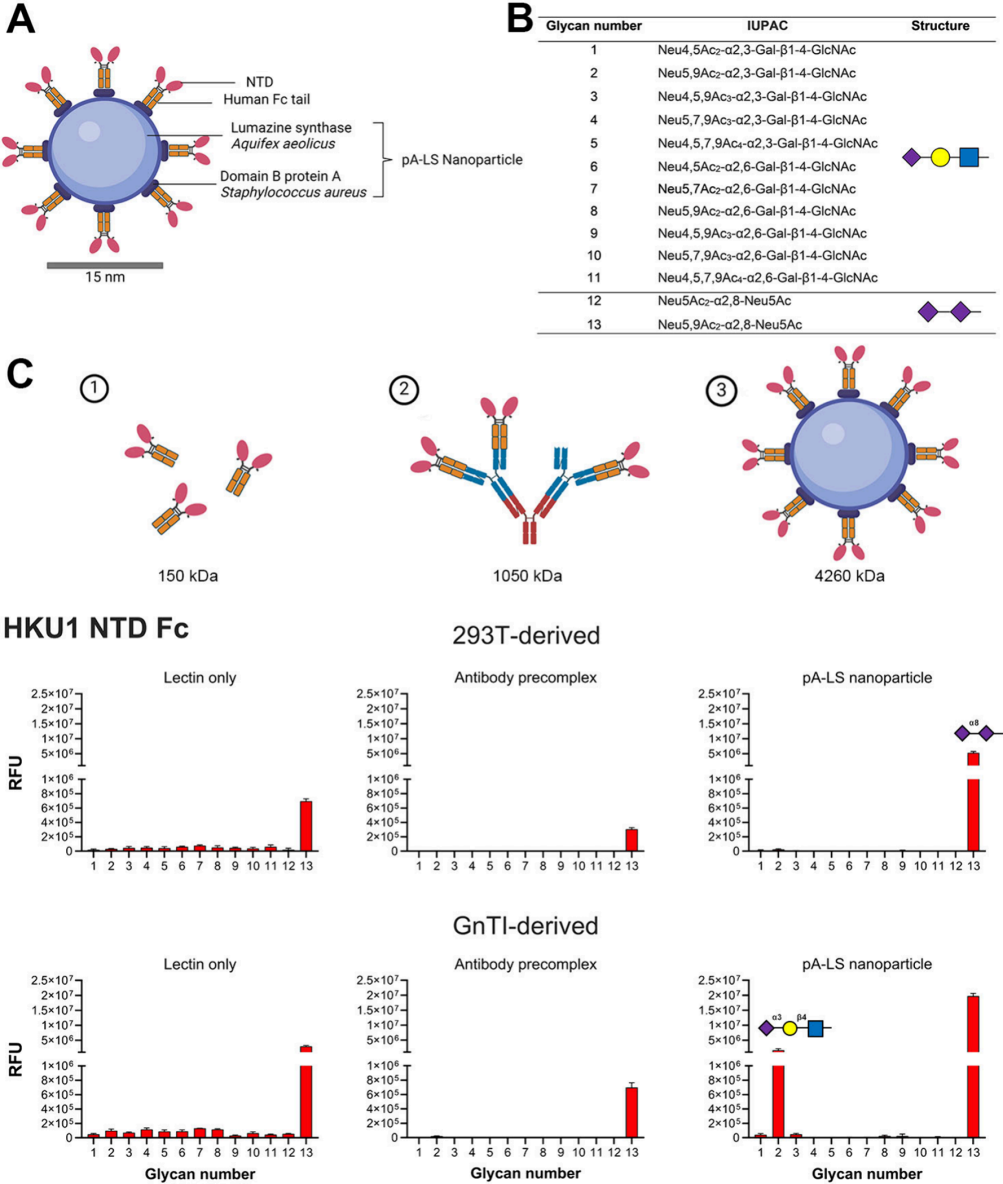
Multimerization-dependent receptor probing. (A) HKU1 NTD Fc chimera presented on a 60-mer pA-LS nanoparticle. (B) List of glycans imprinted on the microarray. (C) A glycan microarray was used for evaluation of receptor specificity of (1) HKU1 NTD Fc, (2) precomplexation using antibodies, and (3) conjugation on a 60-mer pA-LS nanoparticle. Predicted molecular weights of the lectin only, antibody precomplex, and pA-LS nanoparticle are shown. For the antibody precomplex, molecular weight was predicted for a complex consisting of 4 lectins, 2 primary antibodies, and 1 secondary antibody. The molecular weight of the pA-LS system was predicted for a 60-mer pA-LS to which 18 HKU1 NTD Fc dimers can bind. HKU1 NTD Fc derived from HEK293T cells displayed receptor specificity toward 9-*O*-acetylated α2-8 linked disialylated structures as lectin only, precomplexed with antibodies, or presented on an NP. HKU1 NTD Fc derived from HEK293S GnTI^–^ cells recognized 9-*O*-acetylated α2-8-linked disialylated structures when used as lectin only or antibody precomplexed, and an additional α2-3-linked Sia LacNAc structure is bound when HKU1 NTD is presented on an NP. See also Tables S2–S4.

Several naturally occurring nanoparticle platforms enable mimicry of pathogens by displaying multiple viral envelope proteins in a spherical form.^35−37^ Here, we employ a self-assembling NP scaffold based on Lumazine Synthase (LS) from *Aquifex aeolicus* fused with domain B of protein A (pA) from *Staphylococcus aureus*.^37,38^ The pA part from the pA-LS scaffold (60-mer) enables high-affinity interaction with antibody Fc regions; thus, to present HCoV-HKU1 S1-NTD on the NP scaffold, HCoV-HKU1 NTD Fc chimeras were expressed and combined with pA-LS (Figure 1A). HCoV-HKU1 NTD, expressed in 293T and used as a lectin only, recognizes the 9-*O*-acetylated α2-8-linked disialic acid (#**13**) as previously described (Figure 1B,C).^10,15^ The same binding pattern was observed when this protein was expressed as a trimer, or precomplexed using antibodies, or conjugated to a nanoparticle (Figure 1C and Figure S1).

Expressing HCoV-HKU1 S1^A^ proteins with complete glycosylation results in lower binding affinity compared to proteins containing high-mannose glycans.^15^ To overcome the low binding affinity of complex glycosylated HCoV-HKU1 NTD proteins in receptor binding, HEK293S GnTI^–^ cells were employed for the expression of HCoV-HKU1 proteins. These cells lack *N*-acetylglucosaminyltransferase I, which processes high mannose to hybrid and complex *N*-glycans;^34^ therefore, proteins expressed by these cells will contain high-mannose glycans. Non- and precomplexed NTD Fc chimeras did not recognize additional structures; however, with a multivalent presentation using pA-LS NP, binding to a 9-*O*-acetylated α2-3 Sia LacNAc structure was observed (Figure 1B and Table S1). The expression of HCoV-HKU1 NTD in GnTI-deficient cells and multivalent presentation on pA-LS NP enabled characterization of previously undetected receptor binding specificity to an abundant terminal epitope.

### HKU1 Recognizes *O*-Acetylated Sia on Different Glycoconjugates

Previously, disialylated gangliosides were indicated as a cellular receptor for HCoV-HKU1, as ST8Sia1 was required to facilitate binding toward nonsusceptible HEK293T cells.^10^ The ST8Sia1 enzyme transfers Sia to an α2-3-linked Sia in GM3, thus generating α2-8-linked disialylated GD3. Sias with an α2-8-linkage are formed through eight different α2-8-sialyltransferases (ST8Sia1-8)^39^ and the acceptor substrate (i.e., glycolipid, *N*- or *O*-glycan) varies per ST8Sia. Thus, the contribution of *N*- and *O*-glycans in receptor binding was not assessed due to the exclusion of ST8Sias that transfer Sia to these glycan cores.

**Figure 2:**
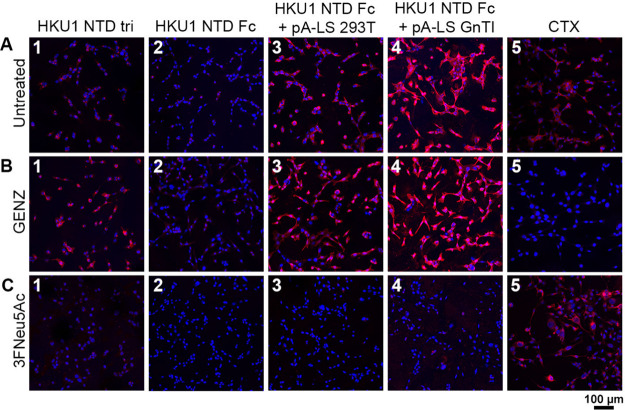
Glycolipid-independent receptor specificity. Trimeric HKU1 NTD, HKU1 NTD Fc, and HKU1 NTD Fc + pA-LS (293T-derived and GnTI^–^-derived) binding to (A) untreated, (B) GENZ-treated, and (C) 3Fneu5Ac-treated RD cells. Utilization of a glycolipid inhibitor (GENZ-123346) did not result in a reduction of the fluorescence intensity for HKU1 NTD (B1–B4). Binding of HKU1 NTD was decreased when cells were treated with 3FNeu5Ac (C1, C3, C4). CTX binding (A5) was decreased after the addition GENZ (B5), while 3FNeu5Ac treatment did not influence binding (C5). Each picture is representative of a biological triplicate.

To assess the contribution of *N*- and *O*-glycans in addition to glycolipids, the rhabdomyosarcoma (RD) cancer cell line was employed as this cell line is susceptible for live HCoV-HKU1 virus.^40^ HCoV-HKU1 NTD was expressed as a trimer using a GCN4 trimerization domain (HCoV-HKU1 NTD tri)^27^ or as an Fc chimera (HCoV-HKU1 NTD Fc). For both trimeric and Fc HCoV-HKU1 NTD variants, binding was observed on RD cells (Figure 2A1,A2). The fluorescence intensity of trimeric NTD (Figure 2A1) in comparison to NTD Fc (Figure 2A2) appeared to be higher related to additional receptor binding sites in a trimer versus in a dimer.

Multivalent presentation of HCoV-HKU1 NTD Fc on a pA-LS NP (Figure 2A3,A4) increased the fluorescence intensity. When comparing HEK293T-derived (Figure 2A3) and HEK293S GnTI^–^-derived (Figure 2A4) proteins, a notable increase in signal was observed for the GnTI^–^-derived HCoV-HKU1 NTD. This boost in binding is probably related to the observation that an additional 9-*O*-acetylated α2-3-linked Sia LacNAc structure is bound. Treatment of RD cells with a glycolipid inhibitor (GENZ-123346) was performed to characterize the contribution of sialoglycolipids to receptor binding (Figure 2B1–B5). Inhibition of glycolipid synthesis was verified using cholera toxin (CTX, Figure 2B5), but no decrease in binding for HCoV-HKU1 NTD was observed (Figure 2B1–B4). In fact, the level of binding of the HKU1 NTD trimer seemed to increase upon glycolipid synthesis inhibition (Figure 2B1). Clearly, *N*- and *O*-sialoglycans can compensate for glycolipid inhibition. To verify that Sias are indeed the determinant for receptor interaction, a sialyltransferase inhibitor (3FNeu5Ac) was employed (Figure 2C1–C5). *Sambucus nigra* lectin (SNA), *Maackia amurensis* lectin II (MAL-II), and *Erythrina cristagalli* lectin (ECA) were used to verify sialic acid inhibition by 3FNeu5Ac (Figure S2). Sialic acid inhibition resulted in a decreased level of HCoV-HKU1 NTD binding (Figure 2C1–C4). In conclusion, we demonstrate glycolipid-independent and verify sialic acid-dependent receptor binding of HCoV-HKU1 NTD. The contribution of different glycoconjugates presented on cell surfaces needs to be taken into consideration to define receptor binding specificity profiles of viruses.

### Redundancy in Receptor Recognition for HKU1 Coronavirus

**Figure 3:**
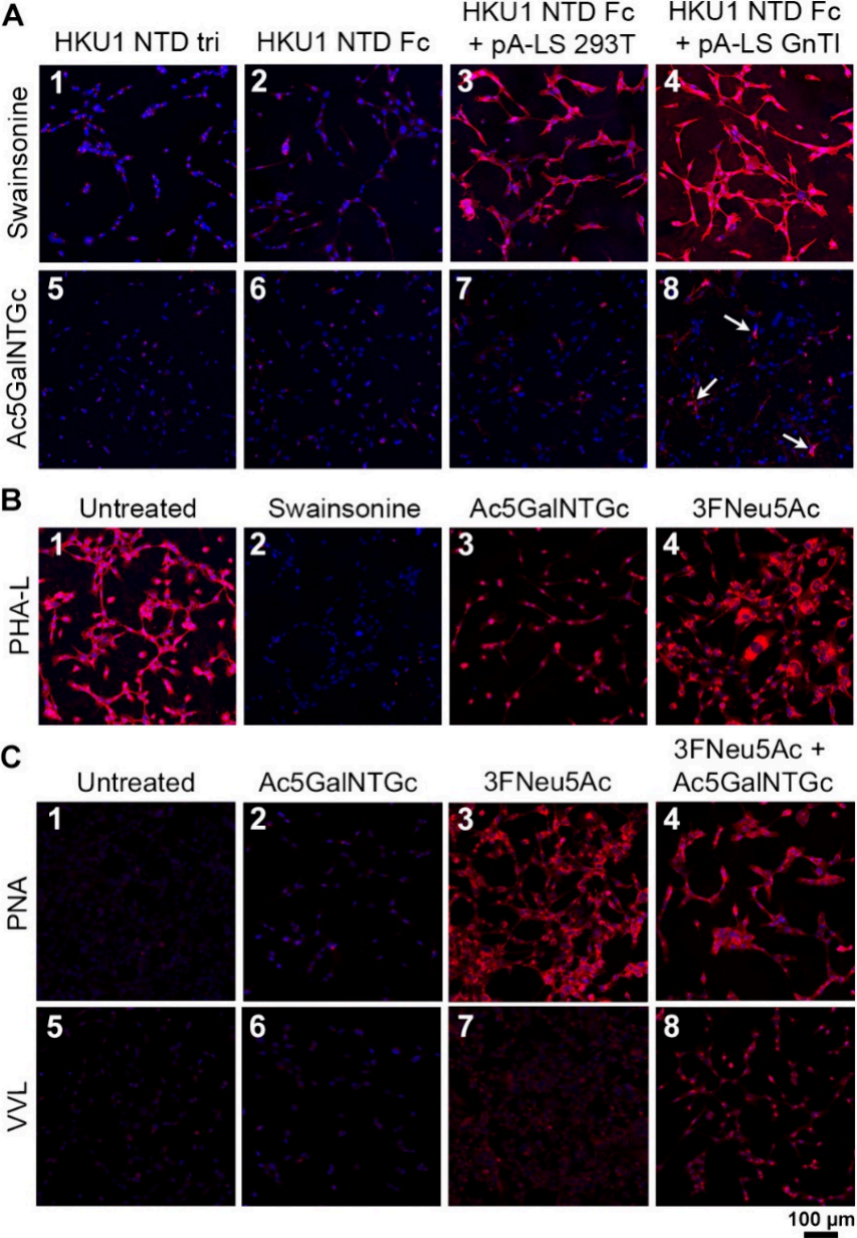
Receptor recognition is independent of the glycan core. Each picture is representative of a biological triplicate. (A) Complex *N*-glycan-independent receptor binding for trimeric HKU1 NTD (A1, A5), HKU1 NTD Fc (A2, A6), and HKU1 NTD Fc + pA-LS (293T-derived (A3, A7) and GnTI^–^-derived (A4, A8)). Receptor binding was not influenced by the addition of a complex *N*-glycan inhibitor (swainsonine; A1–A4). The fluorescence signal was significantly reduced after the use of an *O*-glycan inhibitor (Ac5GalNTGc; A5–A8), albeit spots with high fluorescence intensity were still observed (indicated by arrows; A8). (B) Complex *N*-glycan synthesis inhibition was verified with PHA-L lectin; a reduction of fluorescence intensity was visualized with the use of swainsonine (B2), while Ac5GalNTGc (B3) and 3Fneu5Ac (B4) did not influence PHA-L binding. (C) PNA (C1–C4) and VVL (C5–C8) lectins were used as controls for *O*-glycan truncation after the Ac5GalNTGc treatment.

The sialoglycome on cell surfaces consists of glycolipids and *N*- and *O*-glycans. Glycolipid-independent binding of HCoV-HKU1 NTD warranted further characterization of the *N*- and *O*- glycan contribution in receptor recognition. The synthesis of complex *N*-glycans was inhibited with swainsonine; this compound blocks conversion of hybrid-type to complex-type *N*-glycans by Golgi α-mannosidase II. Inhibition of complex *N*-glycans elaborated that binding of HCoV-HKU1 NTD, as a trimer and (multivalent) Fc chimera, was not dependent on complex *N-*glycosylation (Figure 3A1–A4). The Ac5GalNTGc inhibitor was employed to study the contribution of *O-*glycans as it blocks the transfer of galactose by Core 1 β1,3-galactosyltransferase (C1GalT1) to GalNAcα1-Ser/Thr (Tn-antigen), blocking core 1 *O*-glycan extension.^41^ Although the overall fluorescence intensity was diminished, the Fc chimera and Fc + pA-LS still bound, indicating binding to non-*O*-glycosylated structures (Figure 3A5–A8). Phytohemagglutinin-L (PHA-L) lectin was utilized to assess complex *N*-glycan inhibition, since this lectin recognizes β1-6-branched *N*-linked glycans.^42^ Swainsonine treatment indeed decreased PHA-L binding, while this was not the case with Ac5GalNTGc inhibition (Figure 3B1–B3). Verification of *O*-glycan trimming using Ac5GalNTGc was performed using peanut agglutinin (PNA) and *Vicia villosa* lectin (VVL): these lectins recognize the T- and Tn-antigen, respectively, and an increase in fluorescence intensity indicates *O*-glycan inhibition/trimming. Addition of the inhibitor did not improve the binding of PNA and VVL to RD cells (Figure 3C1,C2,C5,C6), probably due to aberrant sialylation as is common in cancerous cells, like the RD cells employed here, which blocks the binding of these lectins.^43^ The influence of Sia on binding was assessed by utilizing 3FNeu5Ac; the fluorescence signal increased for both PNA and VVL, albeit more significantly for PNA (Figure 3C3,C7). To confirm *O*-glycan trimming, cells were treated with 3FNeu5Ac + Ac5GalNTGc. An increase in intensity concerning 3FNeu5Ac + Ac5GalNTGc-treated cells compared to 3FNeu5Ac-treated cells was observed for PNA and VVL (Figure 3C4,C8); however, this was most pronounced for VVL. Since the fluorescence intensity for PNA and VVL increased upon treating cells with both 3FNeu5Ac and Ac5GalNTGc, we determined that *O*-glycans were trimmed.

**Figure 4:**
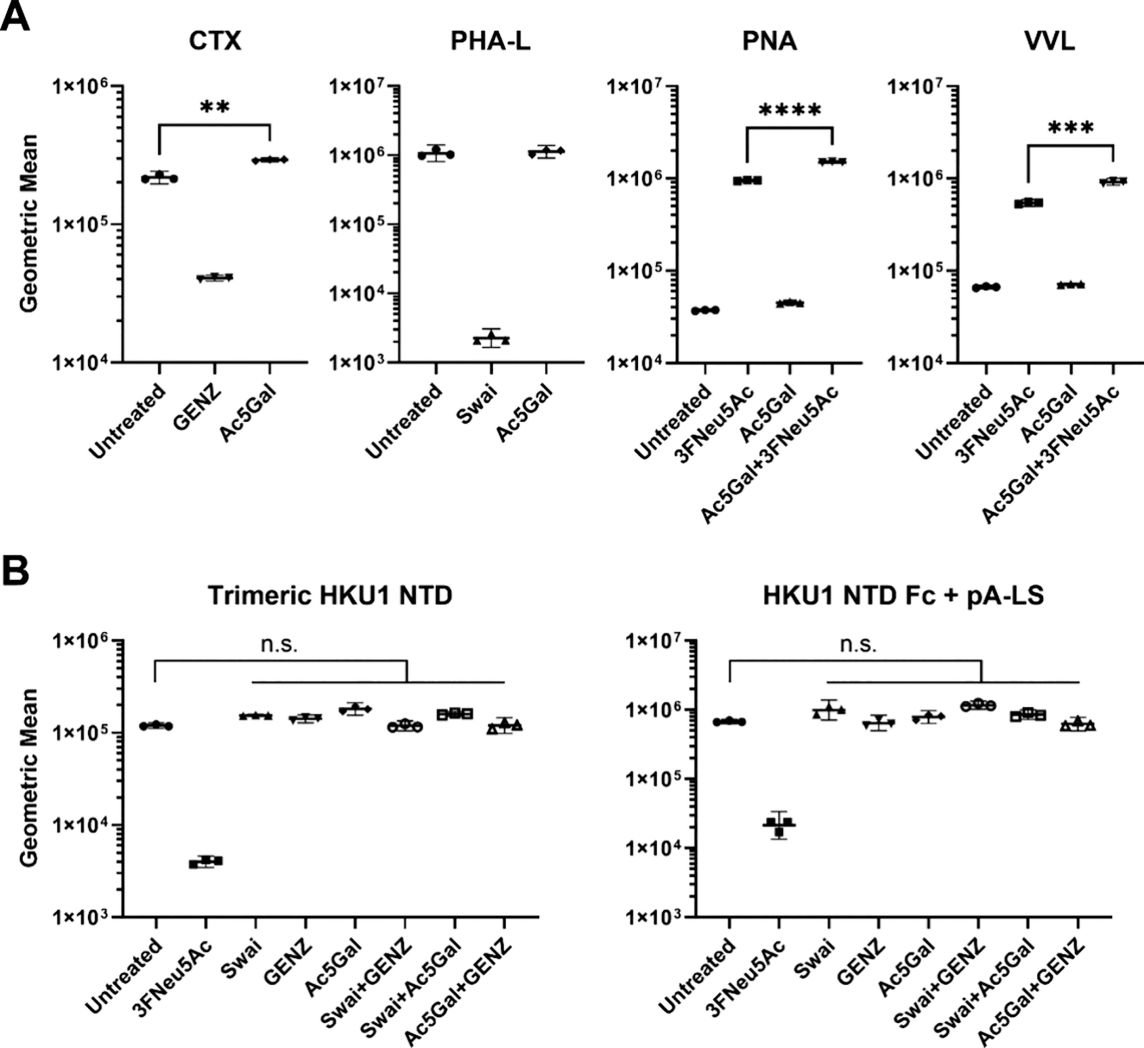
Redundant glycan receptor recognition of HKU1 NTD. Inhibition of glycolipid, complex *N*- and *O*-glycan truncation on RD cells verified using flow cytometry. (A) CTX and PHA-L fluorescence signals decreased after inhibitor treatment. Desialylation with 3FNeu5Ac increased PNA and VVL binding; hereafter, combination of 3FNeu5Ac with Ac5GalNTGc enabled observation and confirmation of *O*-glycan truncation. (B) Receptor recognition of trimeric HKU1 NTD (293T-derived) and HKU1 NTD Fc (GnTI^–^-derived) presented on pA-LS NP was only influenced by desialylation. Data were collected in three independent experiments. The results shown are independent mean values of triplicates obtained in a representative experiment. Standard deviations and *p*-values, calculated by Welch’s *t*-test, are indicated. **p < 0.05, ****p < 0.0001, n.s., not significant. Swai, swainsonine; Ac5Gal, Ac5GalNTGc.

We further characterized core glycan-independent binding using flow cytometry. We first verified the inhibition of glycolipids and complex-type *N*- and *O*-glycans using CTX, PHA-L, PNA, and VVL lectins (Figure 4A). Interestingly, Ac5GalNTGc treatment significantly increased (*p* < 0.05) CTX binding to RD cells. Similar to confocal imaging, the fluorescence intensity for PNA and VVL increased upon treating cells with both 3FNeu5Ac and Ac5GalNTGc (Figure 4A). Trimeric HCoV-HKU1 NTD and NTD Fc + pA-LS binding was decreased by 3FNeu5Ac treatment (Figure 4B). However, although confocal imaging showed an apparent trimeric HKU1 NTD binding increase upon GENZ treatment (Figure 2B1), this increase in binding was not observed by using flow cytometry (Figure 4B). We hypothesized the increase to be due to redundancy, which might not have been strong enough to be observable in flow cytometry. Additionally, treatment with GENZ, swainsonine, and Ac5GalNTGc did not display any dependence on the inhibited glycan core for either trimeric HCoV-HKU1 NTD or HKU1 NTD Fc + pA-LS. To specifically isolate the individual glycan cores, further characterization with a combination of inhibitors, such as swainsonine + GENZ, swainsonine + Ac5GalNTGc, and Ac5GalNTGc + GENZ, was performed. Despite inhibiting a significant portion of the sialoglycome, HCoV-HKU1 NTD binding was maintained, and it is thus fully redundant. This suggests that 9-*O*-acetylated structures are presented on various core glycans and that promiscuous binding is employed by HCoV-HKU1.

### Mucins as a Potential First Point of Contact for HCoV-HKU1

**Figure 5:**
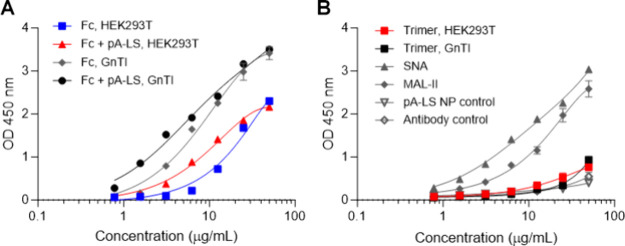
Virolectin binding is α2-8-linked-independent. Experiments were performed in triplicate. Each data point represents the average of the independent mean values. (A) HEK293S GnTI^–^-derived HKU1 NTD Fc and HKU1 NTD Fc + pA-LS displayed increased binding to BSM in comparison with their 293T-derived equivalents. (B) The presence of α2-3-Sia on BSM was confirmed by utilizing MAL-II, while SNA binding confirmed the presence of α2-6-Sia. Trimeric HKU1 NTD (GnTI^–^-derived and 293-derived), pA-LS NP, and antibody control did not bind BSM.

Mucus forms a protective layer of the airway epithelium and serves as the first line of defense.^44,45^ The major macromolecular components of mucus are heavily *O*-glycosylated and *O*-acetylated glycoproteins,^8,44,46,47^ known as mucins. HCoV-HKU1 NTD engages 9-*O*-acetylated Sias, independent from the glycan core. Since mucins play a protective role in the airways against pathogens, we aimed to study the interaction with bovine submaxillary mucin (BSM) as it is heavily modified with *O*-acetylated Sia.^8,46^ Fetuin was used as a control; this 48 kDa molecule does not contain *O-*acetylated structures but is heavily sialylated. Trimeric HCoV-HKU1 NTD and Fc chimeras derived from HEK293T or HEK293S GnTI^–^ cells were analyzed for binding to both substrates. HCoV-HKU1 NTD Fc precomplexed with antibodies or presented on pA-LS NP displayed binding properties similar to those of BSM (Figure 5A), albeit stronger for the NP (Table S5). In contrast, no binding was observed for fetuin, as expected as this substrate does not display 9-*O*-acetylated structures (Figure S3). The trimeric NTD failed to bind both BSM and fetuin (Figure 5B and Figure S3). The difference in binding based on the glycosylation state was pronounced, indicating the necessity of utilizing proteins derived from HEK293S GnTI^–^ cells rather than HEK293T-derived proteins with complex glycosylation. The presence of α2-3- and α2-6-Sia was confirmed with the use of MAL-II (α2-3-Sia, BSM) and SNA lectins (α2-6-Sia, BSM) (Figure 5B and Figure S3). Thus, BSM contains α2-3- and α2-6-linked sialylation and *O*-acetylation while lacking α2-8-disialylated Sias. Importantly, BSM does not contain disialylated structures.^48^ Thus, the presence of 9-*O*-acetylation is the driving factor for glycan binding independent of the glycan core.

## Discussion

Here, we demonstrate that multivalency and the glycosylation state influence glycan binding properties of the HCoV-HKU1 NTD protein, enabling the observation of binding 9-*O*-acetylated α2-3-linked Sia LacNAc. Furthermore, additional biochemical experiments were performed to assess the contribution of glycolipids in NTD-glycan binding, as α2-8-linked Sia is often a terminal epitope of such glycans. Inhibition of glycolipid synthesis did not influence the receptor binding properties of the HCoV-HKU1 NTD, indicating the importance of *N-* and *O-*glycans. Similarly, the inhibition of *N-* or *O-*glycans did not elucidate preferential binding to specific glycan cores (glycolipid, *N-* or *O-*glycan). The utilization of Ac5GalNTGc as an *O-*glycan inhibitor reduced the overall fluorescence intensity using confocal microscopy; however, intense fluorescence was still present in certain locations on the cell, resulting in a robust signal using flow cytometry. We conclude that HCoV-HKU1 NTD binding to 9-*O-*acetylated Sias is independent of the glycan core and recognition of nondisialylated structures, warranting further investigation. We then employed BSM as a ligand, which is a heavily *O-*glycosylated and *O-*acetylated glycoprotein that does not contain α2-8-linked disialylated structures^8,44,46−48^ but mainly α2-3- and some α2-6-linked Sias. The displayed α2-6-linked Sia on BSM is SialylTn to a GalNAc instead of a Gal.^48^ HCoV-HKU1 NTD appeared to interact efficiently with the ligands displayed on BSM. Therefore, only the 9-*O-*acetyl modification on Sia is essential for HCoV-HKU1 glycan binding.

The results demonstrate that glycan recognition is not solely determined by the protein structure as *N*-glycoforms proximal to the Sia-binding pocket influence receptor specificity. It has become increasingly accepted that viral envelope proteins naturally carry immature glycans, specifically at the tip where receptor binding modalities are present.^49−51^ Thus, one might argue that expression in GnTI^–^ cells approximates the receptor binding domain regions. Previous studies showed that proteins derived from HEK293T cells display a heterogeneous *N*-glycome, whereas GnTI^–^-derived proteins display high-mannose *N*-glycan Man-5 only.^32,52,53^ It has been shown that expression of the influenza A virus hemagglutinin in GnTI^–^ cells results in improved Sia binding,^32,33^ and this is probably more widely applicable to sialic acid binding proteins, perhaps due to the avoidance of cis-binding.^54^ Conclusively, the multivalent presentation of viral glycan binding envelope proteins, which do not contain large complex *N*-glycosylation, is ideally suited to determine receptor binding properties.

In this study, we used inhibitors to determine the role of sialic acids, glycolipids, and *N-* and *O-*glycans in HCoV-HKU1 NTD receptor binding. One of these inhibitors is swainsonine, which inhibits Golgi α-mannosidase II and therefore blocks conversion of hybrid-type to complex-type *N*-glycans.^55^ Thus, hybrid-type *N-*glycans can be accumulated on swainsonine-treated cells.^55,56^ Additionally, the lectin PHA-L, which binds β1-6-branched *N*-linked glycans,^42^ was used to verify complex *N-*glycan inhibition. PHA-L does not, however, recognize hybrid-type *N-*glycans.^57^ Therefore, the role of hybrid-type *N-*glycans in HCoV-HKU1 NTD receptor binding is still to be defined. In addition, we did not study the role of each of the eight *N*-linked glycosylation sites present in the HCoV-HKU1 NTD as described previously.^51^ However, using cryo-EM data,^21^ some of these *N*-linked glycosylation sites, specifically N19, N29, N114, N171, and N251, are located closely to the sialoglycan-binding site, which could reduce sialoglycan-binding site accessibility. Introducing loss-of-function mutations to remove each HCoV-HKU1 NTD *N*-linked glycosylation site separately could be used to gather insight on the role of each *N*-glycan in sialoglycan binding.

To assess the contribution of *N*- and *O*-glycans to HCoV-HKU1 NTD glycan binding, we used the RD cancer cell line. Although these cells display an upregulated expression of sialic acids and can be bound by HCoV-HKU1,^40^ this cell system is not a true representation of nature, where HCoV-HKU1 enters the upper and lower respiratory tract cells.^58,59^ To shed more light on HCoV-HKU1 NTD Sia binding, 9-*O*-acetylation of Sia expressed on RD cells should be studied. In addition, as far as we are aware, there are no studies that analyze the presence of 9-*O*-acetylated Sia on spike *N*-glycans. Using MS would require not only a high input of glycans; also, a mixture of glycans is expected to be present on the spike surface, which further complicates measuring 9-*O*-acetylation of *N*-glycans. In addition to the cell system, the glycan microarray approach is not without its drawbacks. Our glycan microarray only contains trisaccharide structures with a Galβ1-4GlcNAc backbone and therefore does not fully represent the complexity of the glycome. Although the Galβ1-4GlcNAc termini are the most abundant in the human respiratory tract,^60^ other terminal modifications like sulfation, fucosylation, LacNAc repeats, and branching are not taken into account, which prompted us to employ metabolic inhibitors of distinct glycan cores in HCoV-HKU1-susceptible cells.

The preferential binding to 9-*O-*acetylated disialic acid raises questions regarding receptor binding and whether the 9-*O-*acetyl α2-3-linked Sia LacNAc ligand would facilitate conformational changes in the spike for S1-CTD exposure.^21^ A recent study showed that this is possible using 9-*O-*acetylated NeuAc only,^22^ yet a 10-fold higher concentration was necessary. A potential explanation is that HCoV-HKU1 could reversibly interact with low affinity/high avidity to 9-*O-*acetyl α2-3-linked Sia-containing molecules (mucins), after which the receptor destroying hemagglutinin esterase activity would release the virion from molecules that do not lead to infection. Hereafter, stronger binding to 9-*O-*acetylated disialic acid on glycolipids located on the cell membrane could enable the transition of S1-CTD into an open state for proteinaceous receptor binding. Glycolipids are in principle an efficient glycan receptor to enter a cell due to their close proximity to the cell membrane, and it has been shown for SARS-CoV-2 that such structures are important for infectivity.^61^

The convergent evolutionary path of HCoV-OC43 and HCoV-HKU1 hemagglutinin esterase activity and the S1-NTD of BCoV, HCoV-OC43, and HCoV-HKU1 engaging *O-*acetylated Sias, with preference to certain Sia linkages, indicate host sialoglycome adaptation for optimal replication and tissue tropism. Comparative sialoglycomics of bovids and humans for differences in *O-*acetylation remain to be identified and could enlighten the receptor binding paradigm of embecoviruses.

## Materials and Methods

### Expression Plasmid Generation

Recombinant HCoV-HKU1 spike protein NTD (GenBank: DQ339101; AA 14-294) was inserted via Gibson assembly using cDNAs encoding codon-optimized open reading frames of a full-length HKU1 spike. HCoV-HKU1 NTD was cloned into the pcDNA5 expression vector, with a C-terminal human IgG1 Fc, a tobacco etch virus (TEV) cleavage site (ENLYFQG), a 6xHis-tag, and a Strep-tag (WSHPQFEK; IBA, Germany).^62^ To generate the trimeric HKU1 NTD expression vector, HKU1 NTD cDNA was cloned into the pCD5 expression vector using Gibson assembly, as previously described.^16^ HCoV-HKU1 NTD was inserted after the signal sequence, in frame with a GCN4 trimerization motif (KQIEDKIEEIESKQKKIENEIARIKK), TEV cleavage site, mOrange2 open reading frame, and a Twin Strep-tag (WSHPQFEKGGGSGGGSGGSAWSHPQFEK; IBA, Germany). For the pA-LS NP expression vector,^37^ domain B of protein A (pA; GenBank: M18264.1, AA 212-270) of *S. aureus* was N-terminally fused to a Strep-tag (WSHPQFEK) and C-terminally fused by a Gly-Ser linker to 6,7-dimethyl-8-ribityllumazine synthase (LS; GenBank: AAC06489.1, AA 1-154) of *A. aeolicus* and constructed in a pUC57 plasmid by GenScript USA, Inc. The pA-LS sequence was ligated into the pCD5 expression vector by using NheI/NotI restriction sites.

### Protein Expression and Purification

Proteins were expressed by transfecting the expression vectors into HEK293T or HEK293S GnTI^–^ cells with polyethylenimine I (PEI) as previously described.^32^ Briefly, cells were grown to 60% confluency in Dulbecco’s modified Eagle medium (DMEM; Gibco) supplemented with 10% fetal calf serum (FCS; Sigma) and 25 units/mL penicillin and 0.025 mg/mL streptomycin (pen/strep; Sigma) in 150 mm cell culture dishes 24 h before transfection. Expression vectors (13.5 μg of HCoV-HKU1 vectors or 40 μg of pA-LS vector per dish) were incubated with PEI in a 1:8 ratio (μg DNA/μg PEI) and DMEM for 20 min before addition to the cells. At 6 h post-transfection, the medium was replaced with 293 SFM II medium (Gibco) supplemented with Primatone (3.0 g/L; Kerry), bicarbonate (3.6 g/L), glucose (2.0 g/L), valproic acid (0.4 g/L), glutaMAX (1%; Gibco), and DMSO (1.5%). Cells were incubated for 5 days at 37 °C and 5% CO_2_ before supernatants were collected. HCoV-HKU1 NTD and pA-LS NP protein expression was analyzed using SDS-PAGE and subsequent western blot on the PVDF membrane (Biorad) using StrepMAB-Classic HRP 1:3000 (2-1509-001, IBA). All proteins were purified using Strep-Tactin Sepharose beads (2-1201-002, IBA) according to the manufacturer's protocol. Proteins were checked on a Coomassie blue-stained SDS-PAGE gel.

### Immunofluorescence Cell Staining

RD cells were grown to 70% confluency on 12 mm coverslips in DMEM supplemented with 10% FCS and 0.25% pen/strep. For depletion of surface glycans, cells were incubated for 72 h with 3FNeu5Ac (5760, Bio-Techne) at 300 μM, Swainsonine (S8195, Sigma) at 10 μM, Ac5GalNTGc (in-house-synthesized) at 80 mM, or GENZ-123346 (T4049, TargetMol) at 5 μM. Functioning of 3FNeu5Ac and GENZ-123346 has been previously validated.^61^ Cells were fixed with 4% paraformaldehyde in phosphate-buffered saline (PBS) for 25 min at RT after which permeabilization was performed using 0.1% Triton in PBS. To present HKU1 NTD Fc chimeras on an NP, NTD Fc proteins were conjugated with pA-LS NP at a 1:1 molar ratio for 1 h at 4 °C. NP-conjugated HKU1 NTD proteins, HKU1 NTD trimers, or Fc chimeras were applied to the coverslips at 50 μg/mL for 1 h at RT. Subsequently, primary StrepMAB-Classic HRP (2-1509-001, IBA) was added to the coverslips for 1 h at RT, followed by a 1 h incubation with secondary goat antimouse IgG Alexa Fluor 555 antibody (Invitrogen) at RT. Both antibodies were added at a 1:500 dilution in PBS. CTX (C1655, Sigma) was applied at a 1:200 dilution in PBS for 1 h at RT. PHA-L (B-1115, Vectorlabs), VVL (B-1235, Vectorlabs), PNA (B-1075, Vectorlabs), SNA (B-1305, Vectorlabs), MAL-II (B-1265, Vectorlabs), and ECA (B-1145–5, Vectorlabs) were applied at 10 μg/mL precomplexed with 2.5 μg/mL streptavidin Alexa Fluor 555 (Thermo Fisher) for 1 h at RT. DAPI (Invitrogen) was used for nuclear staining. All cell stainings were performed three times independently. Samples were imaged on a Leica DMi8 confocal microscope equipped with a 10× HC PL Apo CS2 objective (NA, 0.40). Excitation was achieved with a Diode 405 or white light for the excitation of Alexa 555. A pulsed white laser (80 MHz) was used at 549 nm, and emissions were obtained in the range of 594–627 nm. Laser powers were 10–20% with a gain of a maximum of 200. LAS Application Suite X was used as well as ImageJ for the addition of the scale bar.

### Glycan Microarray

Acetylated structures were printed on glass slides as previously described.^10^ HKU1 NTD proteins at 100 μg/mL were either precomplexed with StrepMAB-Classic HRP (2-1509-001, IBA) and secondary goat antimouse IgG Alexa Fluor 555 (A-21422, Invitrogen) in a 4:2:1 molar ratio or conjugated with pA-LS NP in a 1:1 molar ratio in 50 μL of PBS with 0.1% Tween-20. In the case of pA-LS conjugation, samples were incubated overnight at 4 °C. The mixtures were incubated on the array surface for 90 min in a humidified chamber. Hereafter, the slides were washed successively with PBS-T, PBS, and deionized water. The slides were dried by centrifugation and scanned as described previously.^63^ During processing, the highest and lowest of six replicates were removed, and the mean value and standard deviation of the remaining four replicates were calculated. A list of glycans on the microarray is included in Figure 1B. Glycan microarray details following the MIRAGE guidelines can be found in Tables S2–S4.

### Flow Cytometry Analysis

RD cells growing in a T75 flask were incubated for 72 h with 3FNeu5Ac (no. 5760, Bio-Techne) at 300 μM, Swainsonine (S8195, Sigma) at 10 μM, Ac5GalNTGc (in-house synthesized) at 80 mM, GENZ-123346 (T4049, TargetMol) at 5 μM, or a combination of these inhibitors. Approximately 50,000 cells per well were seeded in a 96-well U-bottom plate. HEK293S GnTI^–^-derived HKU1 NTD Fc at 50 μg/mL was conjugated to pA-LS NP at a 1:1 molar ratio for 1 h on ice. NP-conjugated HKU1 NTD Fc and HEK293T-derived trimeric HKU1 NTD were added to the cells at 50 μg/mL for 1 h on ice. After washing with PBS, the cells were incubated for 1 h at 4 °C with 25 μg/mL StrepMAB-Classic HRP (2-1509-001, IBA), followed by 12.5 μg/mL goat antimouse IgG Alexa Fluor 555 (A-21422, Invitrogen). CTX (C1655, Sigma) was applied to the cells at a 1:200 dilution. PHA-L (30280, Vectorlabs), VVL (B-1235, Vectorlabs), and PNA (30272, Vectorlabs) at 10 μg/mL were applied with 2.5 μg/mL streptavidin Alexa Fluor 555 (Thermo Fisher) for 1 h at 4 °C. Viability staining was performed with ViaKrome 808 viability dye (C36628, Beckman Coulter) 1:10,000 diluted in FACS buffer (d-PBS supplemented with 0.5% bovine serum albumin (BSA) and 2 mM EDTA) for 5 min at 4 °C, followed by centrifugation at 300 rcf for 5 min. After washing with PBS, cells were resuspended in a FACS buffer. Flow cytometry was performed with a CytoFLEX LX (Beckman Coulter). Data were analyzed using FlowJo software and gated as described in Figure S4 to select cells and single cells. Mean fluorescence values for technical triplicates were averaged, and standard deviations were calculated. Significance with *p*-values was tested using Welch’s *t*-test.

### Enzyme-Linked Immunosorbent Assay

Nunc MaxiSORP 96-well plates (Invitrogen) were coated with 10 μg/mL BSM or fetuin in PBS overnight at 4 °C, followed by blocking with 3% BSA in 0.1% PBS-T for 3 h at RT. HKU1 NTD Fc proteins were conjugated with pA-LS NP at a 1:1 molar ratio for 30 min on ice. Trimeric HKU1 NTD, HKU1 NTD Fc, and HKU1 NTD Fc + pA-LS at 50 μg/mL were incubated with StrepMAB-Classic (2-1509-001, IBA) and goat αnti-mouse IgG HRP (31430, Invitrogen) at a 4:2:1 molar ratio for 30 min on ice. As positive controls, SNA (B-1305, VectorLabs) and MAL-II (L-1260, VectorLabs) at 50 μg/mL were precomplexed with rabbit antibiotin HRP (A150-109P, Bethyl) and donkey antirabbit Alexa-fluor 555 (A-31572, Invitrogen) at a 4:2:1 molar ratio. Proteins were added to the plate in a 2-fold serial dilution series and incubated for 1 h at RT. Ultra TMB-ELISA (34028, Thermo Scientific) was added to the plate, and the reaction was stopped after 4 min using 2.5 M H_2_SO_4_. Absorbance was measured at 450 nm in a POLARstar Omega plate reader (BMG Labtech). Geometric mean values for technical triplicates were averaged, and standard deviations were calculated.

### Ac5GalNTGc Synthesis

The synthesis of Ac5GalNTGc was modified from the reported literature.^41^ Briefly, NHS ester of *S*-acetyl-thioglycolic acid (1.2 equiv, 50 mg, 0.22 mmol) was added to a solution of galactosamine (1.0 equiv, 38 mg, 0.18 mmol) and triethylamine (2 equiv, 0.36 mmol, 50 μL) in DMF (4 mL) at RT. This mixture was stirred at RT for 6 h until starting material was invisible on TLC. Then, pyridine (4 mL) and acetic anhydride (2 mL) were added sequentially to the reaction mixture. The reaction was stirred at RT for 24 h, after which the solution was concentrated in vacuo. The residue was taken up in DCM and washed once with 1 M HCl, once with saturated NaHCO_3_, and once with brine. The organic phase was dried with Na_2_SO_4_ and filtrated. The solution was concentrated in vacuo. The residue was purified with silica gel column chromatography and then reverse-phase HPLC (C18) to give the pure compound as an alpha/beta mixture (ratio ∼3:2; 26 mg total; isolated yield, 32%). MS: calc., 463.1148 (C_18_H_25_NO_11_S); observed, 463.1157. Proton and HSQC NMR spectra are shown in Figure S5.

## Supporting Information Available

Table with coronaviruses and their corresponding receptors; glycan microarray details following MIRAGE guidelines; raw glycan microarray data; EC50 values and calculated significance of BSM ELISA; glycan microarray of HKU1 NTD trimer; confocal microscopy pictures verifying sialic acid inhibition on RD cells by 3FNeu5Ac; graphs with fetuin ELISA data; flow cytometry gating strategy; proton and HSQC spectra for Ac5GalNTGc (PDF)

## References

[ref1] Everest H.; Stevenson-Leggett P.; Bailey D.; Bickerton E.; Keep S. Known Cellular and Receptor Interactions of Animal and Human Coronaviruses: A Review. Viruses 2022, 14 (2), 351 10.3390/v14020351. 35215937 PMC8878323

[ref2] Gorbalenya A. E.; Baker S. C.; Baric R. S.; de Groot R. J.; Drosten C.; Gulyaeva A. A.; Haagmans B. L.; Lauber C.; Leontovich A. M.; Neuman B. W.; Penzar D.; Perlman S.; Poon L. L. M.; Samborskiy D. V.; Sidorov I. A.; Sola I.; Ziebuhr J. The species Severe acute respiratory syndrome-related coronavirus: classifying 2019-nCoV and naming it SARS-CoV-2. Nat. Microbiol. 2020, 5 (4), 536–544. 10.1038/s41564-020-0695-z. 32123347 PMC7095448

[ref3] Hulswit R. J.; de Haan C. A.; Bosch B. J. Coronavirus Spike Protein and Tropism Changes. Adv. Virus Res. 2016, 96, 29–57. 10.1016/bs.aivir.2016.08.004. 27712627 PMC7112277

[ref4] Kesheh M. M.; Hosseini P.; Soltani S.; Zandi M. An overview on the seven pathogenic human coronaviruses. Rev. Med. Virol 2022, 32 (2), e2282 10.1002/rmv.2282. 34339073

[ref5] Jackson C. B.; Farzan M.; Chen B.; Choe H. Mechanisms of SARS-CoV-2 entry into cells. Nat. Rev. Mol. Cell Bio 2022, 23 (1), 3–20. 10.1038/s41580-021-00418-x. 34611326 PMC8491763

[ref6] Thompson A. J.; de Vries R. P.; Paulson J. C. Virus recognition of glycan receptors. Curr. Opin Virol 2019, 34, 117–129. 10.1016/j.coviro.2019.01.004. 30849709 PMC6476673

[ref7] Wasik B. R.; Barnard K. N.; Ossiboff R. J.; Khedri Z.; Feng K. H.; Yu H.; Chen X.; Perez D. R.; Varki A.; Parrish C. R. Distribution of O-Acetylated Sialic Acids among Target Host Tissues for Influenza Virus. mSphere 2017, 2 (5), e00379 10.1128/mSphere.00379-16. PMC5588038 28904995

[ref8] Langereis M. A.; Bakkers M. J.; Deng L.; Padler-Karavani V.; Vervoort S. J.; Hulswit R. J.; van Vliet A. L.; Gerwig G. J.; de Poot S. A.; Boot W.; van Ederen A. M.; Heesters B. A.; van der Loos C. M.; van Kuppeveld F. J.; Yu H.; Huizinga E. G.; Chen X.; Varki A.; Kamerling J. P.; de Groot R. J. Complexity and Diversity of the Mammalian Sialome Revealed by Nidovirus Virolectins. Cell Rep 2015, 11 (12), 1966–1978. 10.1016/j.celrep.2015.05.044. 26095364 PMC5292239

[ref9] Wasik B. R.; Barnard K. N.; Parrish C. R. Effects of Sialic Acid Modifications on Virus Binding and Infection. Trends Microbiol 2016, 24 (12), 991–1001. 10.1016/j.tim.2016.07.005. 27491885 PMC5123965

[ref10] Li Z.; Lang Y.; Liu L.; Bunyatov M. I.; Sarmiento A. I.; de Groot R. J.; Boons G. J. Synthetic O-acetylated sialosides facilitate functional receptor identification for human respiratory viruses. Nat. Chem. 2021, 13 (5), 496–503. 10.1038/s41557-021-00655-9. 33753916

[ref11] Stencel-Baerenwald J. E.; Reiss K.; Reiter D. M.; Stehle T.; Dermody T. S. The sweet spot: defining virus-sialic acid interactions. Nat. Rev. Microbiol 2014, 12 (11), 739–749. 10.1038/nrmicro3346. 25263223 PMC4791167

[ref12] Baumann A. M. T.; Bakkers M. J. G.; Buettner F. F. R.; Hartmann M.; Grove M.; Langereis M. A.; de Groot R. J.; Mühlenhoff M. 9-O-Acetylation of sialic acids is catalysed by CASD1 a covalent acetyl-enzyme intermediate. Nat. Commun. 2015, 6, 7673 10.1038/ncomms8673. 26169044 PMC4510713

[ref13] Tortorici M. A.; Walls A. C.; Lang Y.; Wang C.; Li Z.; Koerhuis D.; Boons G. J.; Bosch B. J.; Rey F. A.; de Groot R. J.; Veesler D. Structural basis for human coronavirus attachment to sialic acid receptors. Nat. Struct Mol. Biol. 2019, 26 (6), 481–489. 10.1038/s41594-019-0233-y. 31160783 PMC6554059

[ref14] Petitjean S. J. L.; Chen W.; Koehler M.; Jimmidi R.; Yang J.; Mohammed D.; Juniku B.; Stanifer M. L.; Boulant S.; Vincent S. P.; Alsteens D. Multivalent 9-O-Acetylated-sialic acid glycoclusters as potent inhibitors for SARS-CoV-2 infection. Nat. Commun. 2022, 13 (1), 2564 10.1038/s41467-022-30313-8. 35538121 PMC9091252

[ref15] Hulswit R. J. G.; Lang Y.; Bakkers M. J. G.; Li W.; Li Z.; Schouten A.; Ophorst B.; van Kuppeveld F. J. M.; Boons G. J.; Bosch B. J.; Huizinga E. G.; de Groot R. J. Human coronaviruses OC43 and HKU1 bind to 9-O-acetylated sialic acids via a conserved receptor-binding site in spike protein domain A. Proc. Natl. Acad. Sci. U. S. A. 2019, 116 (7), 2681–2690. 10.1073/pnas.1809667116. 30679277 PMC6377473

[ref16] Tomris I.; Unione L.; Nguyen L.; Zaree P.; Bouwman K. M.; Liu L.; Li Z.; Fok J. A.; Rios Carrasco M.; van der Woude R.; Kimpel A. L. M.; Linthorst M. W.; Kilavuzoglu S. E.; Verpalen E.; Caniels T. G.; Sanders R. W.; Heesters B. A.; Pieters R. J.; Jimenez-Barbero J.; Klassen J. S.; Boons G. J.; de Vries R. P. SARS-CoV-2 Spike N-Terminal Domain Engages 9-O-Acetylated alpha2–8-Linked Sialic Acids. ACS Chem. Biol. 2023, 18 (5), 1180–1191. 10.1021/acschembio.3c00066. 37104622 PMC10178783

[ref17] Forni D.; Cagliani R.; Clerici M.; Sironi M. Molecular Evolution of Human Coronavirus Genomes. Trends Microbiol 2017, 25 (1), 35–48. 10.1016/j.tim.2016.09.001. 27743750 PMC7111218

[ref18] Vijgen L.; Keyaerts E.; Lemey P.; Maes P.; Van Reeth K.; Nauwynck H.; Pensaert M.; Van Ranst M. Evolutionary history of the closely related group 2 coronaviruses: porcine hemagglutinating encephalomyelitis virus, bovine coronavirus, and human coronavirus OC43. J. Virol 2006, 80 (14), 7270–7274. 10.1128/JVI.02675-05. 16809333 PMC1489060

[ref19] Lang Y.; Li W.; Li Z.; Koerhuis D.; van den Burg A. C. S.; Rozemuller E.; Bosch B. J.; van Kuppeveld F. J. M.; Boons G. J.; Huizinga E. G.; van der Schaar H. M.; de Groot R. J. Coronavirus hemagglutinin-esterase and spike proteins coevolve for functional balance and optimal virion avidity. Proc. Natl. Acad. Sci. U. S. A. 2020, 117 (41), 25759–25770. 10.1073/pnas.2006299117. 32994342 PMC7568303

[ref20] Bakkers M. J.; Lang Y.; Feitsma L. J.; Hulswit R. J.; de Poot S. A.; van Vliet A. L.; Margine I.; de Groot-Mijnes J. D.; van Kuppeveld F. J.; Langereis M. A.; Huizinga E. G.; de Groot R. J. Betacoronavirus Adaptation to Humans Involved Progressive Loss of Hemagglutinin-Esterase Lectin Activity. Cell Host Microbe 2017, 21 (3), 356–366. 10.1016/j.chom.2017.02.008. 28279346 PMC7104930

[ref21] Pronker M. F.; Creutznacher R.; Drulyte I.; Hulswit R. J. G.; Li Z.; van Kuppeveld F. J. M.; Snijder J.; Lang Y.; Bosch B. J.; Boons G. J.; Frank M.; de Groot R. J.; Hurdiss D. L. Sialoglycan binding triggers spike opening in a human coronavirus. Nature 2023, 624 (7990), 201–206. 10.1038/s41586-023-06599-z. 37794193 PMC10700143

[ref22] Wang H.; Liu X.; Zhang X.; Zhao Z.; Lu Y.; Pu D.; Zhang Z.; Chen J.; Wang Y.; Li M.; Dong X.; Duan Y.; He Y.; Mao Q.; Guo H.; Sun H.; Zhou Y.; Yang Q.; Gao Y.; Yang X.; Cao H.; Guddat L.; Sun L.; Rao Z.; Yang H. TMPRSS2 and glycan receptors synergistically facilitate coronavirus entry. Cell 2024, 187 (16), 4261–4271. 10.1016/j.cell.2024.06.016. 38964329

[ref23] McCallum M.; Park Y. J.; Stewart C.; Sprouse K. R.; Addetia A.; Brown J.; Tortorici M. A.; Gibson C.; Wong E.; Ieven M.; Telenti A.; Veesler D. Human coronavirus HKU1 recognition of the TMPRSS2 host receptor. Cell 2024, 187 (16), 4231–4245. 10.1016/j.cell.2024.06.006. 38964328 PMC12854727

[ref24] Fernandez I.; Saunders N.; Duquerroy S.; Bolland W. H.; Arbabian A.; Baquero E.; Blanc C.; Lafaye P.; Haouz A.; Buchrieser J.; Schwartz O.; Rey F. A. Structural basis of TMPRSS2 zymogen activation and recognition by the HKU1 seasonal coronavirus. Cell 2024, 187 (16), 4246–4260. 10.1016/j.cell.2024.06.007. 38964326

[ref25] Gestwicki J. E.; Cairo C. W.; Strong L. E.; Oetjen K. A.; Kiessling L. L. Influencing receptor-ligand binding mechanisms with multivalent ligand architecture. J. Am. Chem. Soc. 2002, 124 (50), 14922–14933. 10.1021/ja027184x. 12475334

[ref26] Sieben C.; Sezgin E.; Eggeling C.; Manley S. Influenza A viruses use multivalent sialic acid clusters for cell binding and receptor activation. PLoS Pathog 2020, 16 (7), e1008656 10.1371/journal.ppat.1008656. 32639985 PMC7371231

[ref27] Nemanichvili N.; Tomris I.; Turner H. L.; McBride R.; Grant O. C.; van der Woude R.; Aldosari M. H.; Pieters R. J.; Woods R. J.; Paulson J. C.; Boons G. J.; Ward A. B.; Verheije M. H.; de Vries R. P. Fluorescent trimeric hemagglutinins reveal multivalent receptor binding properties. J. Mol. Biol. 2019, 431, 842–856. 10.1016/j.jmb.2018.12.014. 30597163 PMC6397626

[ref28] Srinivasan A.; Viswanathan K.; Raman R.; Chandrasekaran A.; Raguram S.; Tumpey T. M.; Sasisekharan V.; Sasisekharan R. Quantitative biochemical rationale for differences in transmissibility of 1918 pandemic influenza A viruses. Proc. Natl. Acad. Sci. U. S. A. 2008, 105 (8), 2800–2805. 10.1073/pnas.0711963105. 18287068 PMC2268540

[ref29] Doll T. A.; Raman S.; Dey R.; Burkhard P. Nanoscale assemblies and their biomedical applications. J. R Soc. Interface 2013, 10 (80), 20120740 10.1098/rsif.2012.0740. 23303217 PMC3565727

[ref30] Bale J. B.; Gonen S.; Liu Y.; Sheffler W.; Ellis D.; Thomas C.; Cascio D.; Yeates T. O.; Gonen T.; King N. P.; Baker D. Accurate design of megadalton-scale two-component icosahedral protein complexes. Science 2016, 353 (6297), 389–394. 10.1126/science.aaf8818. 27463675 PMC5485857

[ref31] Brinkkemper M.; Sliepen K. Nanoparticle Vaccines for Inducing HIV-1 Neutralizing Antibodies. Vaccines 2019, 7 (3), 76 10.3390/vaccines7030076. 31362378 PMC6789800

[ref32] de Vries R. P.; de Vries E.; Bosch B. J.; de Groot R. J.; Rottier P. J.; de Haan C. A. The influenza A virus hemagglutinin glycosylation state affects receptor-binding specificity. Virology 2010, 403 (1), 17–25. 10.1016/j.virol.2010.03.047. 20441997

[ref33] Chen J. R.; Yu Y. H.; Tseng Y. C.; Chiang W. L.; Chiang M. F.; Ko Y. A.; Chiu Y. K.; Ma H. H.; Wu C. Y.; Jan J. T.; Lin K. I.; Ma C.; Wong C. H. Vaccination of monoglycosylated hemagglutinin induces cross-strain protection against influenza virus infections. Proc. Natl. Acad. Sci. U. S. A. 2014, 111 (7), 2476–2481. 10.1073/pnas.1323954111. 24469815 PMC3932897

[ref34] Reeves P. J.; Callewaert N.; Contreras R.; Khorana H. G. Structure and function in rhodopsin: high-level expression of rhodopsin with restricted and homogeneous N-glycosylation by a tetracycline-inducible N-acetylglucosaminyltransferase I-negative HEK293S stable mammalian cell line. Proc. Natl. Acad. Sci. U. S. A. 2002, 99 (21), 13419–13424. 10.1073/pnas.212519299. 12370423 PMC129688

[ref35] Heald-Sargent T.; Gallagher T. Ready, set, fuse! The coronavirus spike protein and acquisition of fusion competence. Viruses 2012, 4 (4), 557–580. 10.3390/v4040557. 22590686 PMC3347323

[ref36] Brinkkemper M.; Veth T. S.; Brouwer P. J. M.; Turner H.; Poniman M.; Burger J. A.; Bouhuijs J. H.; Olijhoek W.; Bontjer I.; Snitselaar J. L.; Caniels T. G.; van der Linden C. A.; Ravichandran R.; Villaudy J.; van der Velden Y. U.; Sliepen K.; van Gils M. J.; Ward A. B.; King N. P.; Heck A. J. R.; Sanders R. W. Co-display of diverse spike proteins on nanoparticles broadens sarbecovirus neutralizing antibody responses. iScience 2022, 25 (12), 105649 10.1016/j.isci.2022.105649. 36439375 PMC9678814

[ref37] Li W.; Hulswit R. J. G.; Widjaja I.; Raj V. S.; McBride R.; Peng W.; Widagdo W.; Tortorici M. A.; van Dieren B.; Lang Y.; van Lent J. W. M.; Paulson J. C.; de Haan C. A. M.; de Groot R. J.; van Kuppeveld F. J. M.; Haagmans B. L.; Bosch B. J. Identification of sialic acid-binding function for the Middle East respiratory syndrome coronavirus spike glycoprotein. Proc. Natl. Acad. Sci. U. S. A. 2017, 114 (40), E8508–E8517. 10.1073/pnas.1712592114. 28923942 PMC5635925

[ref38] Park Y. J.; Walls A. C.; Wang Z.; Sauer M. M.; Li W.; Tortorici M. A.; Bosch B. J.; DiMaio F.; Veesler D. Structures of MERS-CoV spike glycoprotein in complex with sialoside attachment receptors. Nat. Struct Mol. Biol. 2019, 26 (12), 1151–1157. 10.1038/s41594-019-0334-7. 31792450 PMC7097669

[ref39] Harduin-Lepers A.; Petit D.; Mollicone R.; Delannoy P.; Petit J. M.; Oriol R. Evolutionary history of the alpha2,8-sialyltransferase (ST8Sia) gene family: Tandem duplications in early deuterostomes explain most of the diversity found in the vertebrate ST8Sia genes. Bmc Evol Biol. 2008, 8, 258 10.1186/1471-2148-8-258. 18811928 PMC2564942

[ref40] Huang X. C.; Dong W. J.; Milewska A.; Golda A.; Qi Y. H.; Zhu Q. K.; Marasco W. A.; Baric R. S.; Sims A. C.; Pyrc K.; Li W. H.; Sui J. H. Human Coronavirus HKU1 Spike Protein Uses O-Acetylated Sialic Acid as an Attachment Receptor Determinant and Employs Hemagglutinin-Esterase Protein as a Receptor-Destroying Enzyme. J. Virol 2015, 89 (14), 7202–7213. 10.1128/JVI.00854-15. 25926653 PMC4473545

[ref41] Wang S. S.; Solar V. D.; Yu X.; Antonopoulos A.; Friedman A. E.; Agarwal K.; Garg M.; Ahmed S. M.; Addhya A.; Nasirikenari M.; Lau J. T.; Dell A.; Haslam S. M.; Sampathkumar S. G.; Neelamegham S. Efficient inhibition of O-glycan biosynthesis using the hexosamine analog Ac(5)GalNTGc. Cell Chem. Biol. 2021, 28 (5), 699–710. 10.1016/j.chembiol.2021.01.017. 33609441 PMC8140985

[ref42] Cummings R. D.; Kornfeld S. Characterization of the structural determinants required for the high affinity interaction of asparagine-linked oligosaccharides with immobilized Phaseolus vulgaris leukoagglutinating and erythroagglutinating lectins. J. Biol. Chem. 1982, 257 (19), 11230–11234. 10.1016/S0021-9258(18)33746-3. 7118880

[ref43] Bull C.; Boltje T. J.; Wassink M.; de Graaf A. M.; van Delft F. L.; den Brok M. H.; Adema G. J. Targeting aberrant sialylation in cancer cells using a fluorinated sialic acid analog impairs adhesion, migration, and in vivo tumor growth. Mol. Cancer Ther 2013, 12 (10), 1935–1946. 10.1158/1535-7163.MCT-13-0279. 23974695

[ref44] Ridley C.; Thornton D. J. Mucins: the frontline defence of the lung. Biochem. Soc. Trans. 2018, 46 (5), 1099–1106. 10.1042/BST20170402. 30154090 PMC6195635

[ref45] Wardzala C. L.; Wood A. M.; Belnap D. M.; Kramer J. R. Mucins Inhibit Coronavirus Infection in a Glycan-Dependent Manner. ACS Cent Sci. 2022, 8 (3), 351–360. 10.1021/acscentsci.1c01369. 35345395 PMC8864775

[ref46] Feuerbaum S.; Saile N.; Pohlentz G.; Muthing J.; Schmidt H. De-O-Acetylation of mucin-derived sialic acids by recombinant NanS-p esterases of Escherichia coli O157:H7 strain EDL933. Int. J. Med. Microbiol 2018, 308 (8), 1113–1120. 10.1016/j.ijmm.2018.10.001. 30340996 PMC7106450

[ref47] Denneny E.; Sahota J.; Beatson R.; Thornton D.; Burchell J.; Porter J. Mucins and their receptors in chronic lung disease. Clin Transl Immunol 2020, 9 (3), e01120 10.1002/cti2.1120. PMC7077995 32194962

[ref48] Vos G. M.; Hooijschuur K. C.; Li Z.; Fjeldsted J.; Klein C.; de Vries R. P.; Torano J. S.; Boons G. J. Sialic acid O-acetylation patterns and glycosidic linkage type determination by ion mobility-mass spectrometry. Nat. Commun. 2023, 14 (1), 6795 10.1038/s41467-023-42575-x. 37880209 PMC10600165

[ref49] Thompson A. J.; Cao L.; Ma Y.; Wang X.; Diedrich J. K.; Kikuchi C.; Willis S.; Worth C.; McBride R.; Yates J. R. 3rd; Paulson J. C. Human Influenza Virus Hemagglutinins Contain Conserved Oligomannose N-Linked Glycans Allowing Potent Neutralization by Lectins. Cell Host Microbe 2020, 27 (5), 725–735. 10.1016/j.chom.2020.03.009. 32298658 PMC7158820

[ref50] Behrens A. J.; Crispin M. Structural principles controlling HIV envelope glycosylation. Curr. Opin Struct Biol. 2017, 44, 125–133. 10.1016/j.sbi.2017.03.008. 28363124 PMC5513759

[ref51] Watanabe Y.; Berndsen Z. T.; Raghwani J.; Seabright G. E.; Allen J. D.; Pybus O. G.; McLellan J. S.; Wilson I. A.; Bowden T. A.; Ward A. B.; Crispin M. Vulnerabilities in coronavirus glycan shields despite extensive glycosylation. Nat. Commun. 2020, 11 (1), 2688 10.1038/s41467-020-16567-0. 32461612 PMC7253482

[ref52] Allen J. D.; Ivory D. P.; Song S. G.; He W. T.; Capozzola T.; Yong P.; Burton D. R.; Andrabi R.; Crispin M. The diversity of the glycan shield of sarbecoviruses related to SARS-CoV-2. Cell Rep 2023, 42 (4), 112307 10.1016/j.celrep.2023.112307. 36972173 PMC10015101

[ref53] Wang C. C.; Chen J. R.; Tseng Y. C.; Hsu C. H.; Hung Y. F.; Chen S. W.; Chen C. M.; Khoo K. H.; Cheng T. J.; Cheng Y. S.; Jan J. T.; Wu C. Y.; Ma C.; Wong C. H. Glycans on influenza hemagglutinin affect receptor binding and immune response. Proc. Natl. Acad. Sci. U. S. A. 2009, 106 (43), 18137–18142. 10.1073/pnas.0909696106. 19822741 PMC2775302

[ref54] Dhar C.; Sasmal A.; Diaz S.; Verhagen A.; Yu H.; Li W.; Chen X.; Varki A. Are sialic acids involved in COVID-19 pathogenesis?. Glycobiology 2021, 31 (9), 1068–1071. 10.1093/glycob/cwab063. 34192318 PMC8344891

[ref55] Tulsiani D. R.; Touster O. Swainsonine causes the production of hybrid glycoproteins by human skin fibroblasts and rat liver Golgi preparations. J. Biol. Chem. 1983, 258 (12), 7578–7585. 10.1016/S0021-9258(18)32217-8. 6408079

[ref56] Moremen K. W. Golgi alpha-mannosidase II deficiency in vertebrate systems: implications for asparagine-linked oligosaccharide processing in mammals. Biochim. Biophys. Acta 2002, 1573 (3), 225–235. 10.1016/S0304-4165(02)00388-4. 12417404

[ref57] Hall M. K.; Weidner D. A.; Zhu Y.; Dayal S.; Whitman A. A.; Schwalbe R. A. Predominant Expression of Hybrid Glycans Has Distinct Cellular Roles Relative to Complex and Oligomannose Glycans. Int. J. Mol. Sci. 2016, 17 (6), 925 10.3390/ijms17060925. 27304954 PMC4926458

[ref58] Pyrc K.; Sims A. C.; Dijkman R.; Jebbink M.; Long C.; Deming D.; Donaldson E.; Vabret A.; Baric R.; van der Hoek L.; Pickles R. Culturing the unculturable: human coronavirus HKU1 infects, replicates, and produces progeny virions in human ciliated airway epithelial cell cultures. J. Virol 2010, 84 (21), 11255–11263. 10.1128/JVI.00947-10. 20719951 PMC2953148

[ref59] Woo P. C.; Lau S. K.; Chu C. M.; Chan K. H.; Tsoi H. W.; Huang Y.; Wong B. H.; Poon R. W.; Cai J. J.; Luk W. K.; Poon L. L.; Wong S. S.; Guan Y.; Peiris J. S.; Yuen K. Y. Characterization and complete genome sequence of a novel coronavirus, coronavirus HKU1, from patients with pneumonia. J. Virol 2005, 79 (2), 884–895. 10.1128/JVI.79.2.884-895.2005. 15613317 PMC538593

[ref60] Walther T.; Karamanska R.; Chan R. W.; Chan M. C.; Jia N.; Air G.; Hopton C.; Wong M. P.; Dell A.; Malik Peiris J. S.; Haslam S. M.; Nicholls J. M. Glycomic analysis of human respiratory tract tissues and correlation with influenza virus infection. PLoS Pathog 2013, 9 (3), e1003223 10.1371/journal.ppat.1003223. 23516363 PMC3597497

[ref61] Nguyen L.; McCord K. A.; Bui D. T.; Bouwman K. M.; Kitova E. N.; Elaish M.; Kumawat D.; Daskhan G. C.; Tomris I.; Han L.; Chopra P.; Yang T. J.; Willows S. D.; Mason A. L.; Mahal L. K.; Lowary T. L.; West L. J.; Hsu S. D.; Hobman T.; Tompkins S. M.; Boons G. J.; de Vries R. P.; Macauley M. S.; Klassen J. S. Sialic acid-containing glycolipids mediate binding and viral entry of SARS-CoV-2. Nat. Chem. Biol. 2022, 18, 81–90. 10.1038/s41589-021-00924-1. 34754101 PMC12434308

[ref62] Rodrigues E.; Jung J.; Park H.; Loo C.; Soukhtehzari S.; Kitova E. N.; Mozaneh F.; Daskhan G.; Schmidt E. N.; Aghanya V.; Sarkar S.; Streith L.; St. Laurent C. D.; Nguyen L.; Julien J. P.; West L. J.; Williams K. C.; Klassen J. S.; Macauley M. S. A versatile soluble siglec scaffold for sensitive and quantitative detection of glycan ligands. Nat. Commun. 2020, 11 (1), 5091 10.1038/s41467-020-18907-6. 33037195 PMC7547722

[ref63] Broszeit F.; van Beek R. J.; Unione L.; Bestebroer T. M.; Chapla D.; Yang J. Y.; Moremen K. W.; Herfst S.; Fouchier R. A. M.; de Vries R. P.; Boons G. J. Glycan remodeled erythrocytes facilitate antigenic characterization of recent A/H3N2 influenza viruses. Nat. Commun. 2021, 12 (1), 5449 10.1038/s41467-021-25713-1. 34521834 PMC8440751

