# Supplementary material for: The HCoV-HKU1 N-Terminal Domain Binds a Wide Range of 9-O-Acetylated Sialic Acids Presented on Different Glycan Cores
Source: ACS Infect Dis. 2024 Oct 12;10(11):3880–90. doi: 10.1021/acsinfecdis.4c00488 (PMC11555679; doi:10.1021/acsinfecdis.4c00488)
Supplement: Supplementary file 1 — id4c00488_si_001.pdf [file id4c00488_si_001.pdf]

Supporting Information

The HCoV-HKU1 N-terminal domain binds a wide range of 9-*O*-acetylated sialic acids presented on different glycan cores

Ilhan Tomris[1#], Anne L.M. Kimpel[1#], Ruonan Liang[1], Roosmarijn van der Woude[1], Geert-Jan P.H. Boons[1,2], Zeshi Li[1] and Robert P. de Vries[1*]

[1] Department of Chemical Biology & Drug Discovery, Utrecht Institute for Pharmaceutical Sciences, Utrecht University, 3584 CG Utrecht, The Netherlands

[2] Complex Carbohydrate Research Center, University of Georgia, 315 Riverbend Road, Athens, Georgia 30602, United States.

[#] These authors contributed equally to this work

[*] for correspondence: r.vries@uu.nl

Table S1. Coronaviruses and their receptors.

Genus	Name[a]	Receptor[b] S1-NTD	Receptor[b] S1-CTD
Alphacoronavirus	HCoV-229E	unknown	hAPN (1)
	HCoV-NL63	unknown	ACE2, heparan sulfate proteoglycans (2, 3)
Betacoronavirus	HCoV-HKU1	9-*O*-acetylated sialic acid (4)	TMPRSS2 (5)
	HCoV-OC43	9-*O*-acetylated sialic acid (4, 6)	unknown
	MERS-CoV	Sialoglycans (7, 8)	DPP4 (9)
	SARS-CoV-1	unknown	ACE2, heparan sulfate proteoglycans (10, 11)
	SARS-CoV-2	9-*O*-acetylated sialic acid (12)	ACE2, heparan sulfate proteoglycans, sialic acids (13-15)

Notes: [a]HCoV, human coronavirus; MERS-CoV, Middle East respiratory syndrome coronavirus; SARS-CoV, severe acute respiratory syndrome coronavirus. [b]hAPN, human aminopeptidase N; ACE2, angiotensin-converting enzyme 2; TMPRSS2, transmembrane serine protease 2; DPP4, dipeptidylpeptidase 4

Table S2. **Glycan Microarray details according to the MIRAGE Glycan Microarray Guidelines.**

**1. Sample: Glycan Binding Sample**	
Description of the sample	HKU1 NTD proteins were expressed and prepared as described in the method section of the main text.
Sample modification	HKU1 NTD Fc proteins were either not modified, precomplexed with antibodies, or conjugated with pA-LS nanoparticle.<br>Antibody precomplexed HKU1 NTD Fc samples were precomplexed in a 4:2:1 molar ratio with StrepMAB-Classic HRP (IBA) and secondary goat anti-mouse IgG Alexa Fluor 555 (Invitrogen) overnight at 4°C.<br>pA-LS nanoparticle conjugated HKU1 NTD Fc samples were incubated overnight with pA-LS in a 1:1 molar ratio at 4°C.
Assay protocol	HKU1 NTD proteins (100 µg/mL) were incubated with 50 µg/mL StrepMAB-Classic HRP and 25 µg/mL secondary goat anti-mouse IgG Alexa Fluor 555 in 50 µL PBS-T for 15 min on ice and incubated on the array for 90 min at room temperature. Slides were washed with PBS-T, PBS, and deionized water, followed by centrifugation and scanning.
**2. Glycan Library**	
Glycan description for defined glycans	The in-house glycan microarray consisted of 13 defined glycans (Figure 1B). All printed glycans were previously characterized (16).
**3. Printing surfaces**	
Description of surface	SuperStreptavidin Microarray Substrate Slides
Manufacturer	ArrayIt Inc
Covalent Immobilization	Streptavidin-coat for biotinylated glycans

**4. Arrayer**	
Description of arrayer	Non-contact microarray printer, sciFLEXARRAYER S3, Scienion Inc.
Dispensing mechanism	Non-contact, one nozzle
Glycan deposition	400 pL of 100 μM, 40 fmol, 6 replicates
Printing conditions	100 μM printing concentration, 20 °C, 50% humidity, blocking with TSM binding buffer (20 mM Tris·HCl, pH 7, 150 mM NaCl, 2 mM $CaCl_2$ and 2 mM $MgCl_2$, 0.05% Tween-20, 1% BSA) for 1h at 4°C. Slides were rinsed with DI water.
**5. Glycan Microarray**	
Array layout	24 subarrays (3 x 8) were printed per slide.
Glycan identification	The in-house glycan microarray consisted of 13 defined glycans (Figure 1B).
Quality control	Quality control included analyses with HKU1 Fc proteins.
**6. Detector and Data Processing**	
Scanning hardware	Innopsys Innoscan 7200
Scanner settings	Scanning resolution: 5 mm / pixel<br>Laser channel: Iterative scans at 532 nm<br>PMT: 100<br>Scan power: 10% (532 nm), The lower laserpowers were used to avoid overexposure.
Image analysis software	GenePix Pro 7 software
Data processing	Fluorescent intensity from the images, highest and lowest value removed from 6 replicates, total intensities are plotted as mean +/- SD.
**7. Glycan Microarray Data Presentation**	
Data presentation	The microarray binding results are in Figure 1C and Supplementary Tables S3 and S4. Binding results are presented as bar graphs with bars representing the averaged mean relative fluorescence units of each glycan. Error bars represent standard deviation.
**8. Interpretation and Conclusion from Microarray Data**	
Data interpretation	Images were analyzed using GenePix Pro 7 software. The data were further processed with Microsoft Excel and plotted with GraphPad Prism 7.
Conclusions	HKU1 NTD proteins bind 9-*O*-acetylated α2-8-linked disialylated structures. pA-LS conjugated HKU1 NTD Fc binds an α2-3-linked Sia LacNAc structure.

Table S3. Tabulated data of 293T-derived HKU1 NTD Fc receptor binding as shown in Figure 1C. Mean and standard deviation (SD) of four replicates are shown.

	293T-derived HKU1 NTD Fc					
	Lectin only		Antibody precomplex		pA-LS nanoparticle	
Compound	Mean	SD	Mean	SD	Mean	SD
1	21486	8362,5	1205	1423,12	12422,25	7251,14
2	31277,25	5864,4	5151,75	2097,28	24807,5	8212,23
3	45822,25	18762,14	1	0	4055	4920,22
4	49434,75	14643,86	3142,75	2897,15	859,25	1486,53
5	43045,75	19761,98	1	0	1	0
6	60340	9278,01	1	0	1	0
7	77330	8598,96	1	0	1	0
8	52108,25	23284,48	1	0	1	0
9	46260,5	9964,38	1	0	9754,5	6163,54
10	38947	14544,89	1	0	1676	2901,19
11	60233	27098,09	160	275,4	1100,25	1903,96
12	20972,25	20379,41	166,75	287,09	1	0
13	697053,5	32860,48	305485	21244,1	5304605,5	433701,92

Table S4. Tabulated data of GnTI$^-$-derived HKU1 NTD Fc receptor binding as shown in Figure 1C. Mean and standard deviation (SD) of four replicates are shown.

	GnTI$^-$-derived HKU1 NTD Fc					
	Lectin only		Antibody precomplex		pA-LS nanoparticle	
Compound	Mean	SD	Mean	SD	Mean	SD
1	49609,75	11175,71	1	0	42824,75	15521,55
2	98664,75	23484,74	21629,75	2221,52	1693645,75	470040,88
3	71668,5	9828,45	788,25	1363,56	49177,5	12123,87
4	117707,75	19281,18	276,5	477,18	1759,25	3045,38
5	88450,25	22158,96	1	0	625	1080,8
6	89873,5	19646,75	1	0	3304,25	5721,4
7	131387	2919	1	0	3065,25	5307,44
8	116908,5	8525,5	1	0	23962,75	11318,59
9	31221	7780,51	1	0	25287	26820,56
10	66005,75	17592,12	1	0	1	0
11	43909,75	9816,79	1	0	5669,25	9817,7
12	55908	7744,27	1	0	1	0
13	2977175,5	266865,68	698223	65169,37	1,9760953e+007	872338,65

Table S5. EC50 values of HKU1 NTD Fc binding to BSM. EC50 values were based on BSM ELISA data (Figure 5A). A significant difference in HEK293T-derived HKU1 NTD Fc + pA-LS binding to BSM was observed in comparison to HEK293T-derived HKU1 NTD Fc.

Protein	EC50	p-value
Fc, HEK293T	19.064	<0.0001
Fc + pA-LS, HEK293T	9.262	
Fc, GnTI$^-$	7.819	n.s.
Fc + pA-LS, GnTI$^-$	6.786	

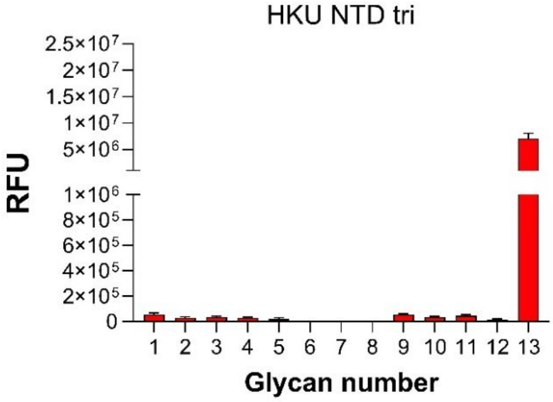

Fig. S1. Glycan microarray analysis of HKU1 NTD trimer receptor-binding specificity. HKU1 NTD trimer derived from HEK293S GnTI$^-$ cells displayed receptor specificity towards 9-*O*-acetylated α2-8 linked disialylated structures (#13). A list of glycans imprinted on the microarray can be found in Figure 1B.

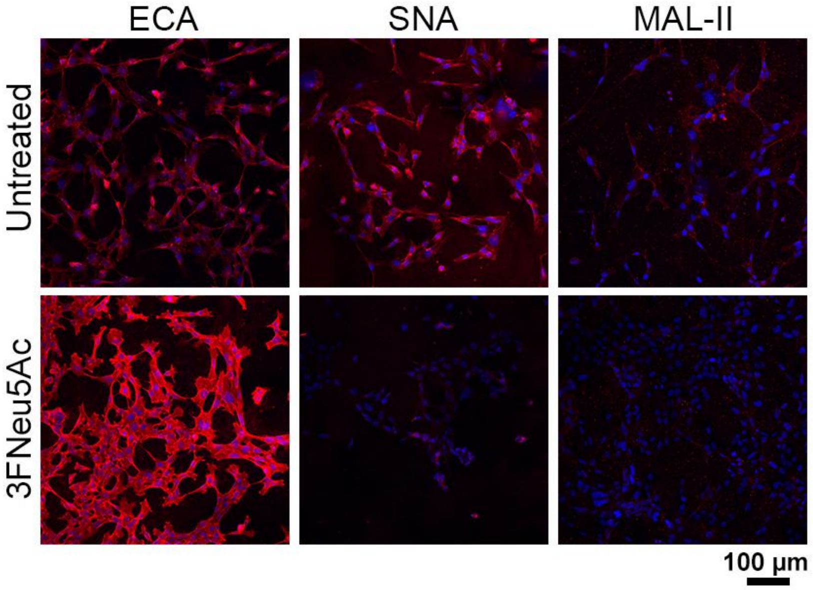

Fig. S2. Confocal microscopy verification of sialic acid inhibition on RD cells by sialyltransferase inhibitor 3Fneu5Ac. Binding of ECA, which binds non-sialylated structures, was increased upon 3Fneu5Ac treatment of RD cells. Binding of sialic acid-binding lectins SNA (α2-6 Sia) and MAL-II (α2-3 Sia) was decreased upon 3FNeu5Ac treatment of RD cells.

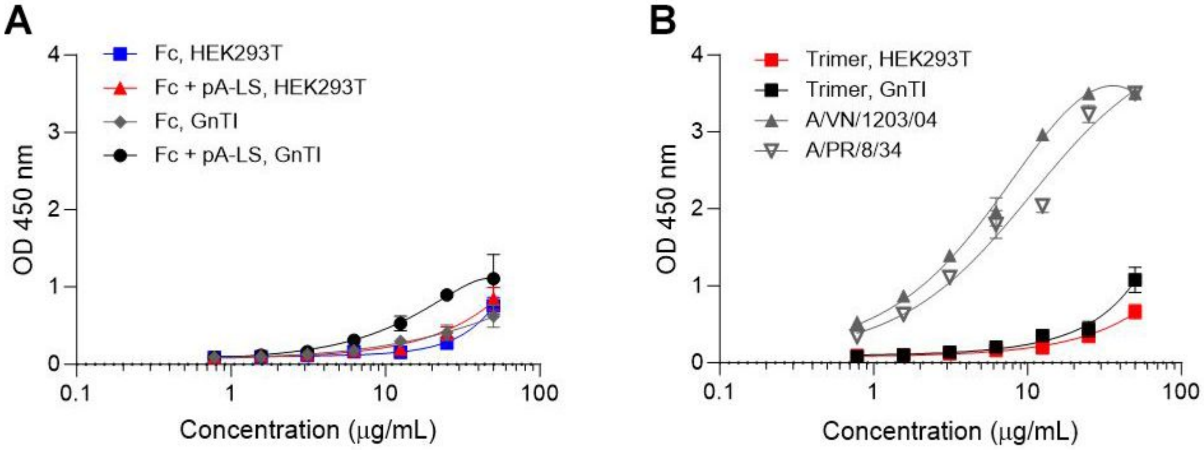

Fig. S3. O-acetylation dependent virolectin binding. (A) HKU1 NTD Fc and HKU1 NTD Fc + pA-LS (GnTI⁻-derived and HEK293T-derived) did not display binding to fetuin. **(B)** Presence of sialic acids on fetuin was confirmed by using A/Vietnam/1203/2004 (A/VN/1203/04) and A/Puerto Rico/8/1934 (A/PR/8/34). Trimeric HKU1 NTD (GnTI⁻-derived and HEK293T-derived) did not bind fetuin.

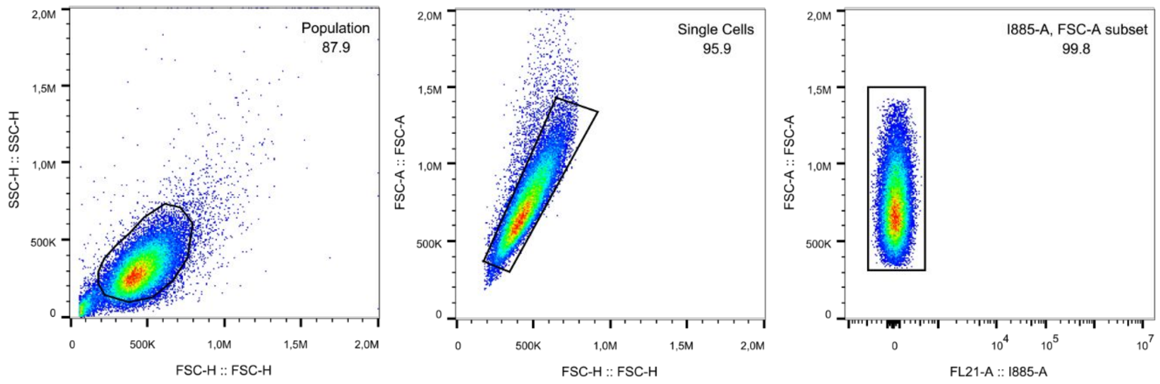

Fig. S4. Gating strategies for flow cytometry. Gating was performed to select for cell population, followed by singlets and then live-cells using ViaKrome 808 viability dye.

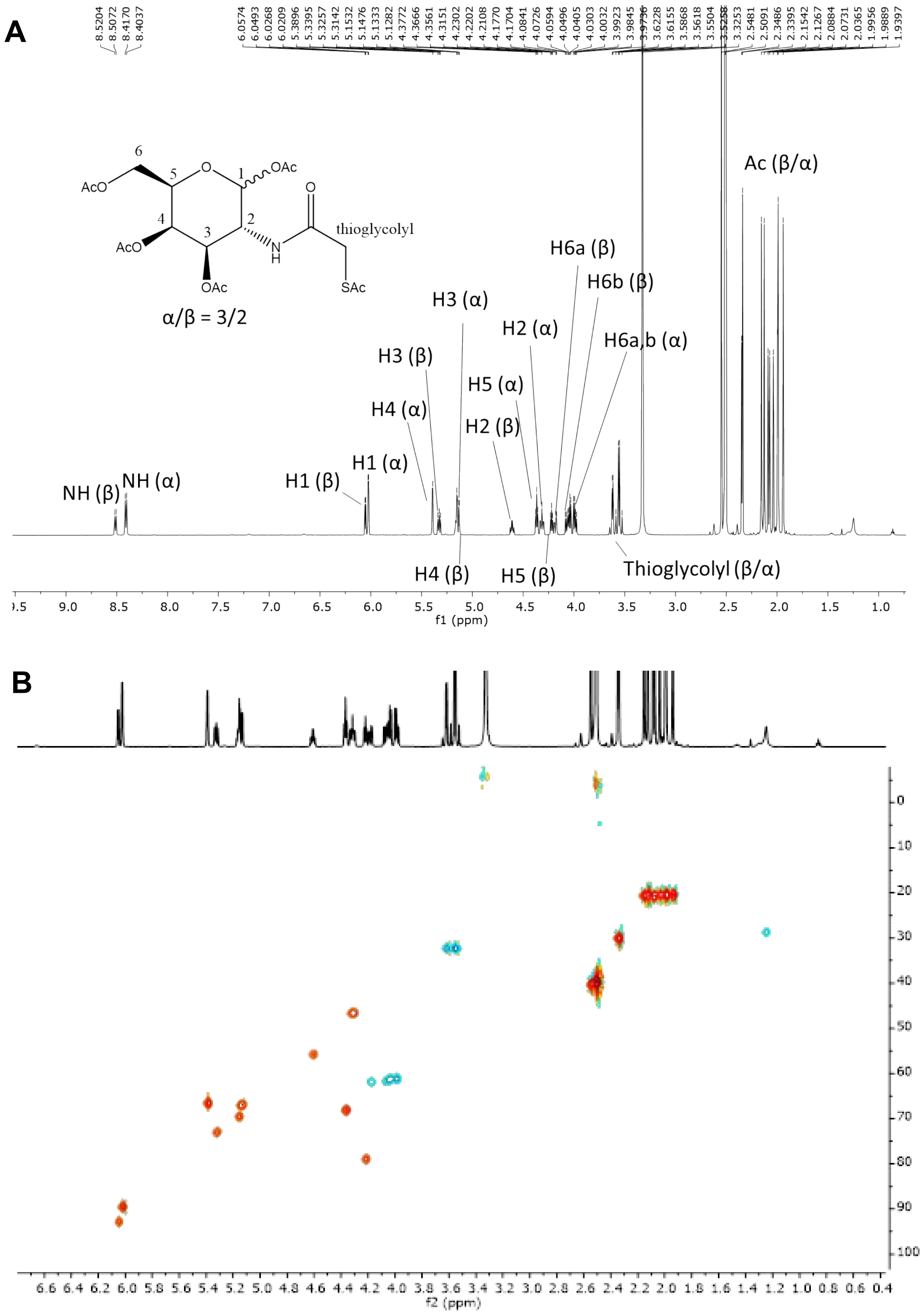

Fig. S5. Proton and HSQC analyses for Ac5GalNTGc. (A) ^{1}H-NMR spectrum (600 MHz, DMSO-d_6) with signal assignments. **(B)** HSQC spectrum (600 MHz, DMSO-d_6).

References

(1) Yeager, C. L.; Ashmun, R. A.; Williams, R. K.; Cardellichio, C. B.; Shapiro, L. H.; Look, A. T.; Holmes, K. V. Human Aminopeptidase-N Is a Receptor for Human Coronavirus-229e. *Nature* **1992**, *357* (6377), 420-422. DOI: DOI 10.1038/357420a0.
(2) Hofmann, H.; Pyrc, K.; van der Hoek, L.; Geier, M.; Berkhout, B.; Pohlmann, S. Human coronavirus NL63 employs the severe acute respiratory syndrome coronavirus receptor for cellular entry. *Proc Natl Acad Sci U S A* **2005**, *102* (22), 7988-7993. DOI: 10.1073/pnas.0409465102.
(3) Milewska, A.; Zarebski, M.; Nowak, P.; Stozek, K.; Potempa, J.; Pyrc, K. Human coronavirus NL63 utilizes heparan sulfate proteoglycans for attachment to target cells. *J Virol* **2014**, *88* (22), 13221-13230. DOI: 10.1128/JVI.02078-14.
(4) Hulswit, R. J. G.; Lang, Y.; Bakkers, M. J. G.; Li, W.; Li, Z.; Schouten, A.; Ophorst, B.; van Kuppeveld, F. J. M.; Boons, G. J.; Bosch, B. J.; Huizinga, E. G.; de Groot, R. J. Human coronaviruses OC43 and HKU1 bind to 9-O-acetylated sialic acids via a conserved receptor-binding site in spike protein domain A. *Proc Natl Acad Sci U S A* **2019**, *116* (7), 2681-2690. DOI: 10.1073/pnas.1809667116.
(5) Saunders, N.; Fernandez, I.; Planchais, C.; Michel, V.; Rajah, M. M.; Baquero Salazar, E.; Postal, J.; Porrot, F.; Guivel-Benhassine, F.; Blanc, C.; Chauveau-Le Friec, G.; Martin, A.; Grzelak, L.; Oktavia, R. M.; Meola, A.; Ahouzi, O.; Hoover-Watson, H.; Prot, M.; Delaune, D.; Cornelissen, M.; Deijs, M.; Meriaux, V.; Mouquet, H.; Simon-Loriere, E.; van der Hoek, L.; Lafaye, P.; Rey, F.; Buchrieser, J.; Schwartz, O. TMPRSS2 is a functional receptor for human coronavirus HKU1. *Nature* **2023**, *624* (7990), 207-214. DOI: 10.1038/s41586-023-06761-7.
(6) Vlasak, R.; Luytjes, W.; Spaan, W.; Palese, P. Human and bovine coronaviruses recognize sialic acid-containing receptors similar to those of influenza C viruses. *Proc Natl Acad Sci U S A* **1988**, *85* (12), 4526-4529. DOI: 10.1073/pnas.85.12.4526.
(7) Li, W.; Hulswit, R. J. G.; Widjaja, I.; Raj, V. S.; McBride, R.; Peng, W.; Widagdo, W.; Tortorici, M. A.; van Dieren, B.; Lang, Y.; van Lent, J. W. M.; Paulson, J. C.; de Haan, C. A. M.; de Groot, R. J.; van Kuppeveld, F. J. M.; Haagmans, B. L.; Bosch, B. J. Identification of sialic acid-binding function for the Middle East respiratory syndrome coronavirus spike glycoprotein. *Proc Natl Acad Sci U S A* **2017**, *114* (40), E8508-E8517. DOI: 10.1073/pnas.1712592114.
(8) Park, Y. J.; Walls, A. C.; Wang, Z.; Sauer, M. M.; Li, W.; Tortorici, M. A.; Bosch, B. J.; DiMaio, F.; Veesler, D. Structures of MERS-CoV spike glycoprotein in complex with sialoside attachment receptors. *Nat Struct Mol Biol* **2019**, *26* (12), 1151-1157. DOI: 10.1038/s41594-019-0334-7.
(9) Raj, V. S.; Mou, H. H.; Smits, S. L.; Dekkers, D. H. W.; Müller, M. A.; Dijkman, R.; Muth, D.; Demmers, J. A. A.; Zaki, A.; Fouchier, R. A. M.; Thiel, V.; Drosten, C.; Rottier, P. J. M.; Osterhaus, A. D. M. E.; Bosch, B. J.; Haagmans, B. L. Dipeptidyl peptidase 4 is a functional receptor for the emerging human coronavirus-EMC. *Nature* **2013**, *495* (7440), 251-254. DOI: 10.1038/nature12005.
(10) Li, W. H.; Moore, M. J.; Vasilieva, N.; Sui, J. H.; Wong, S. K.; Berne, M. A.; Somasundaran, M.; Sullivan, J. L.; Luzuriaga, K.; Greenough, T. C.; Choe, H.; Farzan, M. Angiotensin-converting enzyme 2 is a functional receptor for the SARS coronavirus. *Nature* **2003**, *426* (6965), 450-454. DOI: 10.1038/nature02145.
(11) Lang, J.; Yang, N.; Deng, J.; Liu, K.; Yang, P.; Zhang, G.; Jiang, C. Inhibition of SARS pseudovirus cell entry by lactoferrin binding to heparan sulfate proteoglycans. *PLoS One* **2011**, *6* (8), e23710. DOI: 10.1371/journal.pone.0023710.
(12) Tomris, I.; Unione, L.; Nguyen, L.; Zaree, P.; Bouwman, K. M.; Liu, L.; Li, Z.; Fok, J. A.; Rios Carrasco, M.; van der Woude, R.; Kimpel, A. L. M.; Linthorst, M. W.; Kilavuzoglu, S. E.; Verpalen, E.; Caniels, T. G.; Sanders, R. W.; Heesters, B. A.; Pieters, R. J.; Jimenez-Barbero, J.; Klassen, J. S.; Boons, G. J.; de Vries, R. P. SARS-CoV-2 Spike N-Terminal Domain Engages 9-O-Acetylated alpha2-8-Linked Sialic Acids. *ACS Chem Biol* **2023**, *18* (5), 1180-1191. DOI: 10.1021/acschembio.3c00066.
(13) Zhou, P.; Yang, X. L.; Wang, X. G.; Hu, B.; Zhang, L.; Zhang, W.; Si, H. R.; Zhu, Y.; Li, B.; Huang, C. L.; Chen, H. D.; Chen, J.; Luo, Y.; Guo, H.; Jiang, R. D.; Liu, M. Q.; Chen, Y.; Shen, X. R.; Wang, X.; Zheng, X. S.; Zhao, K.; Chen, Q. J.; Deng, F.; Liu, L. L.; Yan, B.; Zhan, F. X.; Wang, Y. Y.; Xiao, G. F.; Shi,

Z. L. A pneumonia outbreak associated with a new coronavirus of probable bat origin. *Nature* **2020**, *579* (7798), 270-273. DOI: 10.1038/s41586-020-2012-7.
(14) Liu, L.; Chopra, P.; Li, X.; Bouwman, K. M.; Tompkins, S. M.; Wolfert, M. A.; de Vries, R. P.; Boons, G.-J. Heparan Sulfate Proteoglycans as Attachment Factor for SARS-CoV-2. *ACS Cent Sci* **2021**, *7* (6), 1009-1018. DOI: 10.1021/acscentsci.1c00010.
(15) Nguyen, L.; McCord, K. A.; Bui, D. T.; Bouwman, K. M.; Kitova, E. N.; Elaish, M.; Kumawat, D.; Daskhan, G. C.; Tomris, I.; Han, L.; Chopra, P.; Yang, T. J.; Willows, S. D.; Mason, A. L.; Mahal, L. K.; Lowary, T. L.; West, L. J.; Hsu, S. D.; Hobman, T.; Tompkins, S. M.; Boons, G. J.; de Vries, R. P.; Macauley, M. S.; Klassen, J. S. Sialic acid-containing glycolipids mediate binding and viral entry of SARS-CoV-2. *Nat Chem Biol* **2021**, *18*, 81-90. DOI: 10.1038/s41589-021-00924-1.
(16) Li, Z.; Lang, Y.; Liu, L.; Bunyatov, M. I.; Sarmiento, A. I.; de Groot, R. J.; Boons, G. J. Synthetic O-acetylated sialosides facilitate functional receptor identification for human respiratory viruses. *Nat Chem* **2021**, *13* (5), 496-503. DOI: 10.1038/s41557-021-00655-9.
